# Electroreforming of Biomass for Value-Added Products

**DOI:** 10.3390/mi12111405

**Published:** 2021-11-16

**Authors:** Zi Iun Lai, Li Quan Lee, Hong Li

**Affiliations:** 1 School of Mechanical and Aerospace Engineering, Nanyang Technological University, Singapore 639798, Singapore; ZLAI005@e.ntu.edu.sg (Z.I.L.); LIQUAN001@e.ntu.edu.sg (L.Q.L.); 2 Advanced Environmental Biotechnology Centre, Nanyang Environment and Water Research Institute, Nanyang Technological University, 1 Cleantech Loop, Singapore 637141, Singapore; 3 School of Electrical and Electronic Engineering, Nanyang Technological University, Singapore 639798, Singapore; 4 CINTRA CNRS/NTU/THALES, UMI 3288, Research Techno Plaza, Singapore 637553, Singapore

**Keywords:** biomass electroreforming, electrooxidation, electrochemical hydrogenation, cellulose, green hydrogen

## Abstract

Humanity’s overreliance on fossil fuels for chemical and energy production has resulted in uncontrollable carbon emissions that have warranted widespread concern regarding global warming. To address this issue, there is a growing body of research on renewable resources such as biomass, of which cellulose is the most abundant type. In particular, the electrochemical reforming of biomass is especially promising, as it allows greater control over valorization processes and requires milder conditions. Driven by renewable electricity, electroreforming of biomass can be green and sustainable. Moreover, green hydrogen generation can be coupled to anodic biomass electroforming, which has attracted ever-increasing attention. The following review is a summary of recent developments related to electroreforming cellulose and its derivatives (glucose, hydroxymethylfurfural, levulinic acid). The electroreforming of biomass can be achieved on the anode of an electrochemical cell through electrooxidation, as well as on the cathode through electroreduction. Recent advances in the anodic electroreforming of cellulose and cellulose-derived glucose and 5-hydrooxylmethoylfurural (5-HMF) are first summarized. Then, the key achievements in the cathodic electroreforming of cellulose and cellulose-derived 5-HMF and levulinic acid are discussed. Afterward, the emerging research focusing on coupling hydrogen evolution with anodic biomass reforming for the cogeneration of green hydrogen fuel and value-added chemicals is reviewed. The final chapter of this paper provides our perspective on the challenges and future research directions of biomass electroreforming.

## 1. Introduction

Climate change is arguably humanity’s greatest challenge today. In 2019, an Intergovernmental Panel on Climate Change (IPCC) special report established several conditions required to restrict global temperature rise to 1.5 °C above pre-industrial levels. By 2030, carbon emissions would need to be halved, and by 2050, the net carbon released into the atmosphere must be zero [1]. Worryingly, according to IPCC’s 6th assessment report [2], Earth is perilously close to breaching the 1.5 °C goal by 2030. Limiting temperature rise will require extensive effort, but inaction could result in sea level rise of 180 cm by 2100, resulting in damages to the tune of USD 27 trillion per year [3]. With additional temperature rise, considerable increases in heat-related mortality can be expected, with warmer and poorer regions experiencing a disproportionate burden [4]. Consequently, water stress and food shortages could increase in frequency and severity [5,6]. The window for intervening is open but rapidly dwindling [7].

Replacing the burning of fossil fuels as our main energy source will be crucial. The solid and gaseous wastes humankind produces from the consumption of plants (biomass) go into the environment as carbon. The carbon in the air and soil is utilized by plants and turned into biomass and fuel. In this carbon cycle, biomass provides a renewable fuel. The utilization of such biodegradable waste can produce fuel resources and energy which does not result in as much carbon emissions as burning fossil fuel does. The annual biomass production in nature is estimated to be equivalent to 108 billion tons of oil, which is about ten times the world’s energy consumption [8,9]. With a potential 200 billion tons of yield yearly, lignocellulosic biomass is the most abundant kind of natural biomass [10]. It consists primarily of cellulose, which constitutes between 30% and 50% of lignocellulosic biomass [11,12,13]. Cellulose is a crystalline biopolymer, constructed from long chains of D-glucose monomers, and has been isolated from plant matter and studied since the 17th century [14]. However, it was only in recent years that investigations into the valorization of cellulose feedstock became popular because of the growing awareness of green chemistry [15]. More importantly, cellulose is viewed as the significant feedstock component, as it does not compete with dietary sources, for example, edible crops for traditional biofuel production [16]. Methods to reform raw biomass, however, need to overcome cellulose’s recalcitrance. Strong β-1-4-glycosidic linkages between glucose monomers lead to low reactivity, and the network of inter and intramolecular hydrogen bonding between long chains impedes dissolution in chemical solvents. 

Several methods have been developed to break down cellulose, including hydrothermal processes [17], chemical hydrolysis [18], and biological treatments [19]. Hydrothermal processes generally use hot compressed water as the reaction medium to liquefy biomasses, and valuable products such as bio-oils are extracted through organic solvents [20]. Chemical catalysts are similarly employed to depolymerize cellulose, either in an acidic [21] or an alkaline medium [22]. Ethanol is the most common end product from biological treatments and is produced in two steps: cellulase enzymes initially catalyze the hydrolysis of cellulose into short chain sugars, which are then fermented into ethanol using yeasts or bacteria [23]. Each method has its unique benefits and drawbacks in terms of time required, capital costs, chemicals consumed, and control over quality and distribution of products [24,25]. 

**Figure 1:**
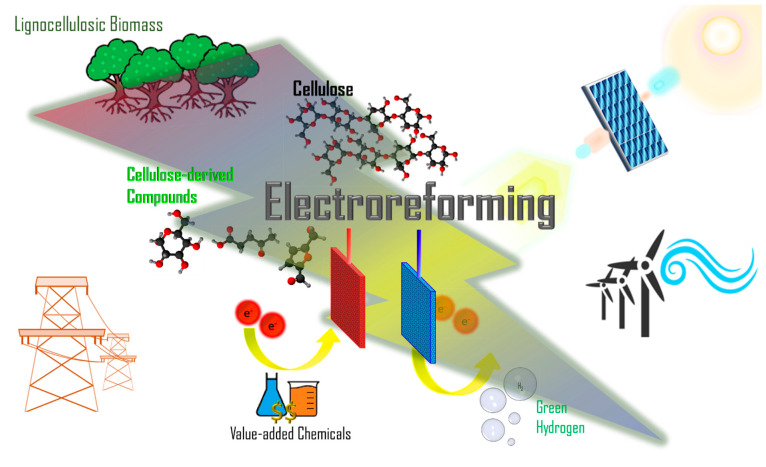
Schematic of biomass electroreforming. The most abundant lignocellulosic biomass is taken as an example. Electroreforming of the major component of lignocellulosic biomass, cellulose, or its derivatives, could offer value-added chemicals and green hydrogen fuel. Electroreforming can be powered by electricity from the grid or renewables, which makes it a promising method of renewable energy storage and green chemistry for a sustainable future.

Electrochemistry represents an effective tool in many important applications. For instance, in wastewater treatment by removal of organic pollutants or drugs (such as antibiotic waste tetracycline hydrochloride) from wastewater [26,27]. In addition, it also represents a promising tool in the valorization of cellulose thanks to its green and sustainable features, as illustrated in Figure 1. Through applying a potential difference across two electrodes, chemical reactions can be driven, with oxidation occurring at the anode (positive electrode) and reduction at the cathode (negative electrode). Two types of desired products—valorized chemicals on the anode and hydrogen gas on the cathode—can be obtained via cellulose electroreforming [28]. Depolymerization and oxidation of cellulose at the anode can produce valuable chemical products, which could reduce reliance on traditional fossil fuel-based and resource-intensive methods of production. Moreover, research on green hydrogen as a fuel source is receiving much attention because combusting hydrogen does not produce pollutants or greenhouse gases, and hydrogen has a high specific energy density [29,30]. Cellulose electroreforming requires catalysts, which can be homogeneous (dispersed in solution) or heterogeneous (often anchored on electrode). In most cases, the electrolyte is generated by dissolving cellulose in sodium hydroxide (NaOH) via the freeze–thaw method first reported in 1998 [31]. Dissolution allows for greater access to active sites on β-1-4-glycosidic linkages [32]. When these linkages are fully broken, cellulose can be depolymerized into its monomer, glucose.

**Figure 2:**
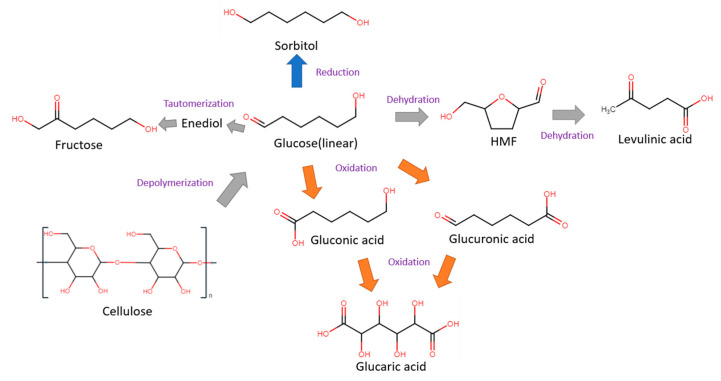
Cellulose, glucose and its derivatives.

Glucose is a simple monosaccharide with molecular formula C_6_H_12_O_6_, and is the main source of energy for most life forms on earth. Apart from cellulose, glucose can also be stored in other polymeric forms, such as starch in plants or glycogen in animals. Glucose can be electrochemically converted to gluconic, glucaric and levulinic acids, as well as 5-HMF. These products can be further valorized into other valuable compounds through electrochemical oxidation (anodic reaction) or hydrogenation (cathodic reaction). Some common glucose transformations are shown in Figure 2. 

Herein, a mini review is presented to summarize the electroreforming of cellulose and its derivatives under different pH conditions using various catalysts. Depending on the targeted final products, the electroreforming of cellulose can be achieved via electrooxidation on the anode or electroreduction on the cathode. We will firstly discuss the recent advances in anodic electroreforming followed by cathodic electroreforming of cellulose and its derivatives. We will focus on the heterogenous catalysts employed and electroreformed products. Then, the emerging research interest on coupling hydrogen evolution (on cathode) with anodic biomass reforming is discussed. To conclude this mini review, our perspectives on the challenges and opportunity on biomass electroreforming are presented. 

## 2. Electroreforming of Biomass at the Anode

### 2.1. Cellulose

Cellulose monomers comprise two anhydroglucose rings of C_5_H_10_O_5_. The rings share an ether (C-O-C) bond between carbon-1 of a glucose ring and carbon-4 of the other, also known as a β-1-4-glycosidic bond. Additionally, intramolecular hydrogen bonding between the hydroxyl group and oxygen on adjacent glucose rings straightens and stabilizes cellulose chains. Intermolecular hydrogen bonding between adjoining cellulose chains also promotes stability and forms crystalline structure [33]. Cellulose exists in four polymorphic varieties: I, II, III and VI. Cellulose I will be focused on here, since it is the natural cellulose found in plant matter, and can be used to form other polymorphs. 

The earliest study passing electricity through cellulose was reported in 1947. O’Sullivan investigated passing current through regenerated cellulose with varying salt and moisture contents to understand the conductance properties of cellulose. In 1963, Murphy performed the first electrolysis of cellulose and observed that hydrogen gas was produced at the anode. However, studies of cellulose electrolysis remained relatively scarce and sporadic until the 21st century, when ever-increasing demand for green and sustainable chemistry appeared. 

**Figure 3:**
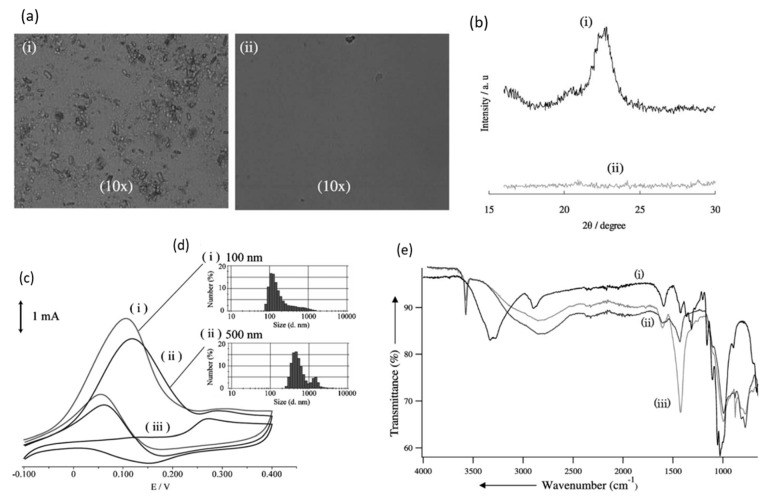
(**a**) Microscopic images before (i) and after (ii) dissolution. (**b**) X-ray diffraction of cellulose before (i) and after (ii) dissolution. (**c**) cyclic voltammogram (vs. Ag/AgCl) of cellulose ball milled to average particle size of 100 nm (i), 500 nm (ii) and without cellulose (iii). (**d**) size distribution after ball milling to 100 nm (i) and 500 nm (ii). (**e**) FTIR scans of cellulose before dissolution (i), after dissolution (ii) and after oxidation (iii). Reprinted with permission from Ref. [34]. Copyright 2010, Electroanalysis.

In 2010, Sugano et al. performed electrooxidation of cellulose at a polycrystalline gold (Au) electrode at a pH of 14 to understand its mechanism [34]. Cellulose powder was first dissolved in NaOH using the freeze–thaw technique, after which the cellulose’s structure was no longer crystalline, suggesting the breakage of intra- and intermolecular hydrogen bonding, which is verified by both microscopic images and X-ray diffraction (XRD) spectroscopy, as presented in Figure 3a,b, respectively. Cyclic voltammetry (CV) revealed two oxidation peaks for dissolved cellulose but not for undissolved cellulose. To explore the effects of particle sizes, ball mill crushing was used to generate cellulose particles of two size ranges, 500 and 100 nm, as shown in Figure 3c,d. Smaller particles led to 13% higher peak current at similar applied potentials, indicating that ball mill pretreatment was efficient in promoting the dissolution of cellulose. Fourier transform infrared imaging (FTIR) scans (Figure 3e) suggested the formation of carboxyl groups, confirming the oxidation of cellulose. To produce direct electricity from oxidation of cellulose nanoparticles, the fuel cell was created and could attain maximum power density of 44 mW/m^2^.

**Figure 4:**
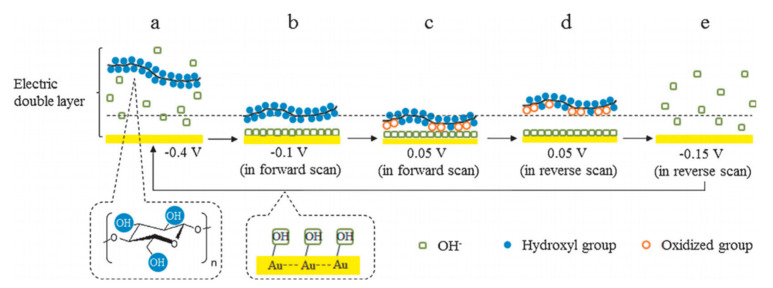
Proposed cellulose oxidation mechanism on electrode surface in alkali media in CV cycle. (**a**) initial state, with cellulose dissolved in alkali solution. (**b**) in forward scan region: adsorption of OH^−^ ions to surface of electrode, cellulose molecule approaches OH-Au active sites. (**c**) oxidation of cellulose at electrode. (**d**) in reverse scan region: desorption of oxidation product and further oxidation of cellulose at revealed electrode surface. (**e**) desorption of OH^−^ ions from electrode surface. Reprinted with permission from Ref. [35]. Copyright 2014, ChemSusChem.

Later, their team studied the electroreforming mechanism in a similar alkaline medium [35]. CV scans suggested that cellulose electrooxidation is irreversible and diffusion-controlled. FTIR was conducted during CV to understand interactions between cellulose and the Au electrode, which suggests that the adsorbed cellulose displaces OH^−^ ions near the electrode surface; and during oxidation, the strength of intermolecular H bonds decreases while that of intramolecular bonds increases. The authors proposed the reaction pathway, as illustrated in Figure 4. Firstly, OH^−^ ions adsorb onto the surface of gold electrodes to form OH-Au sites. These active sites then allow cellulose to adhere electrochemically with Au electrode surface. Cellulose is oxidized and remains adsorbed until reversed potential is applied. The authors hypothesized that the adsorption and desorption of OH^−^ ions play an important catalytic role in cellulose electrooxidation. Nuclear magnetic resonance (NMR) spectra and scanning electron microscope (SEM) imaging suggested the differences in the structure of cellulose after electroreforming; however, the exact products were not identified.

**Figure 5:**
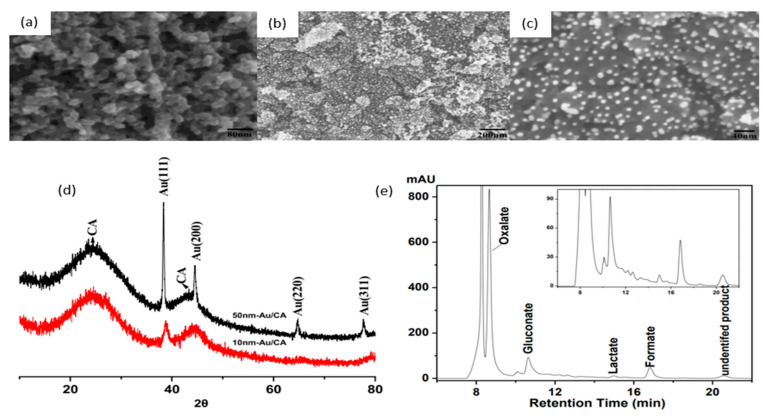
(**a**) SEM image of pretreated CA. (**b**) SEM of 10 nm Au/CA. (**c**) Magnified SEM of 10 nm Au/CA. (**d**) XRD results of 10 nm Au/CA and 50 nm Au/CA electrodes. (**e**) High performance liquid chromatography results of products using 10 nm Au/CA electrodes. Reprinted with permission from Ref. [36]. Copyright 2015, Catalysis.

A notable investigation was conducted in 2016, when Xiao and coworkers reported the use of gold nanoparticles (AuNP) for cellulose electroreforming [36]. Motivated by similar works on cellobiose [37,38], the authors explored the use of nitric acid (HNO_3_)-pretreated carbon aerogel with AuNP as an anode, as shown in Figure 5. Carbon aerogel was fabricated as per a previous report [39], before acid pretreatment. A mixture containing Au precipitates was prepared by reacting HAuCl_4_ and NaB_4_ [40]. Deposition of AuNP onto carbon aerogel was performed by repeatedly dipping aerogel into the mixture and drying it. The anode was used to oxidize cellulose in 0.125 M NaOH (pH of 13.1) with insufflated air. Introduction of oxygen was proposed to speed up the formation of active oxygen species to accelerate oxidation. The authors compared three anodes to study the activities of AuNP with different sizes: 50 nm gold particles on graphite, 50 nm gold particles on carbon aerogel, and 10 nm gold particles on carbon aerogel, as shown in Figure 5a–c. AuNP sizes were calculated from XRD data (Figure 5d) and supported by SEM measurements. Cellulose oxidation was controlled at 10 mA/cm^2^ and products were evaluated with high-performance liquid chromatography (HPLC), as presented in Figure 5e. 

Comparing anodes consisting of 10 and 50 nm AuNP on the same supporting substrate (CA), one can see that the 50 nm NP anode also obtained high cellulose conversion, but selectivity towards gluconate was significantly lower, suggesting that the size of AuNP influences the selectivity of oxidation products. Reducing aeration did not change oxidation products but decreased the rate of reaction. Ten-nanometer gold particles on acid-pretreated carbon aerogel produced gluconic acid at a 67.8% yield with a conversion yield of at least 88.9%.

When comparing anodes with the same 10 nm gold particles on different supporting substrates, the pretreated carbon aerogel supports resulted in better cellulose conversion than that of graphite support. The authors hypothesized that acid pretreatment of CA led to a higher surface area and oxidized surface carbon, favoring the adsorption of cellulose molecules. 

Sugano et al. also attempted to understand cellulose electroreforming mechanism using AuNPs, again in alkaline (pH of 14) conditions [41]. Carbon paper supports were loaded with AuNPs through the chemical precipitation-deposition method. The electrodes were then calcinated at different temperatures (40, 250, and 350 °C). The author observed that calcination at 40 °C led to the formation of Au^+^ (52.5%) and Au^3+^ (30%), while calcinations at higher temperature led to completely metallic AuNPs. Moreover, calcination at 250 °C produced AuNPs with sizes ranging in a narrow band (10–25 nm), whereas a further increased temperature of 350 °C resulted in larger clusters (20–30 nm). CVs were conducted with 1.3 M NaOH against a Ag/AgCl reference, with and without (1 wt%) cellulose present. Anode calcinated at 40 °C displayed no electrocatalytic activity. Moreover, the anode calcinated at 350 °C shows lower electrocatalytic activity than the anode calcinated at 250 ^°^C, indicating that small AuNPs (<25 nm) have higher activity. 

**Figure 6:**
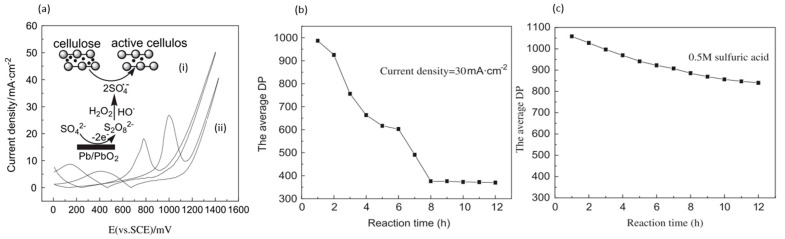
(**a**) CVs (vs. SCE) of 0.5 M sulfuric acid (i) and 0.5 M sulfuric acid with 10 g/L cellulose (ii). (**b**) Decrease in DP over time at 30 mA/cm^2^. (**c**) Decrease in DP over time without applied current. Reprinted with permission from Ref. [42]. Copyright 2011, Polymer Degradation and Stability.

Meng et al. electrolyzed cellulose powder in 0.5 M sulfuric acid (pH of 0.3), rather than in alkali solution, using lead/lead dioxide (Pb/PbO_2_) electrodes [42], as depicted in Figure 6a. Under ambient conditions, with a controlled current density of 30 mA/cm^2^ for 8 h, the degree of polymerization (DP) at average decreased from 1100 to 367. Conversely, the sample that was exposed only to acid had a final DP of 840, as displayed in Figure 6b,c. XRD measurement showed a reduced degree of crystallinity, while FTIR showed lower intensities associated with H and C-O-C bonds without the formation of new groups. A maximum soluble sugar content yield of 2.5% and the highest 5-HMF yield of 1.8% were obtained. 

**Figure 7:**
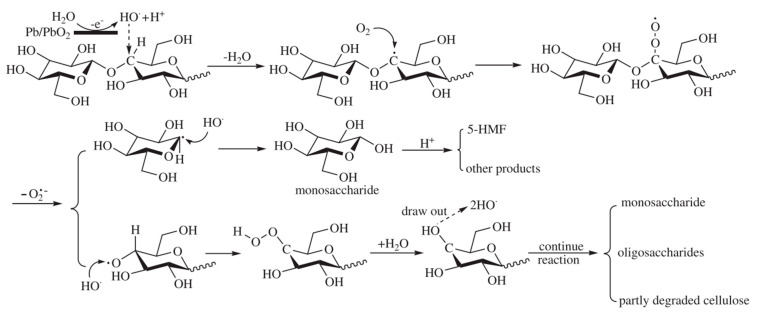
Proposed cellulose depolymerization mechanism in acidic media. Reprinted with permission from Ref. [42]. Copyright 2011, Polymer Degradation and Stability.

Meng and coworkers proposed an electrocatalytic depolymerization process, as presented in Figure 7. Firstly, water molecules lose electrons at the anode to form hydroxyl radicals (OH^−^). Next, the OH^−^ attacks the C-H bond and removes the hydrogen atom from carbon-4 of the glucose unit, leaving a carbon radical (C^−^) in cellulose. C^−^ is then oxidized to become a superoxide radical ($O_{2}^{-}$). Finally, the glycosidic bond is cleaved through the removal of the superoxide radical.

### 2.2. Glucose

Glucose is the most abundant monosaccharide on Earth [43]. Electrolysis of glucose was reported as early as 1866 to produce acetaldehyde; early reports also speculated on the possible formation of alcohol and many other substances [44]. Indeed, studies have revealed a variety of useful products from glucose electroreforming, including 5-HMF, gluconic acid, glucaric acid, gluconolactone and levulinic acid, among others. The synthesis of glucaric acid in particular has been widely acknowledged for its high value. With applications in many industries, glucaric acid has being listed as one of the most value-added chemicals by the US Department of Energy [45,46,47]. However, conventional methods for glucaric acid production often require intense pressures, temperatures and harsh chemicals [48]. Gluconic acid is an intermediate towards the production of glucaric acid, and by itself has other commercial applications [49,50]. Levulinic acid and 5-HMF are platform compounds for producing other useful chemicals [51,52,53]. 

**Figure 8:**
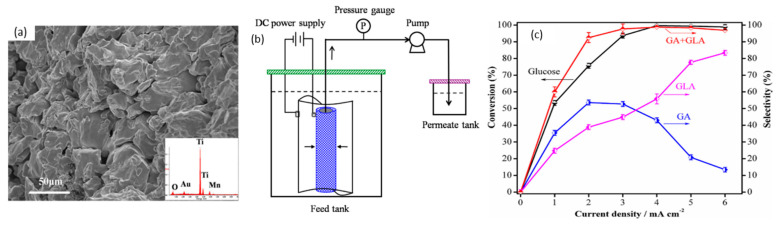
Nano MnO_2_/Ti electrocatalysts in flow-through cells for glucaric acid (GA) and gluconic acid (GLA) production from glucose. (**a**) SEM image of 5% MnO_2_/Ti electrode. (**b**) Schematic diagram of electrocatalytic flow reactor system. (**c**) Products at different current densities (under 5% MnO_2_ loading, glucose concentration of 50.5 mmol/L, pH of 7, 30 °C, 19 min). Reprinted with permission from Ref. [54]. Copyright 2014, Electrochimica Acta.

In 2014, Bin et al. optimized conversion of glucose to glucaric and gluconic acids with nano-manganese dioxide (MnO_2_) loaded tubular porous titanium (Ti) electrodes in a flow-through electrolytic cell, as shown in Figure 8a,b [54]. Interestingly, unlike most other studies, pH was concluded to be less significant in this case—increasing pH from 2 to 10 only changed glucose conversion slightly, from 90% to 93%. Selectivity to gluconic acid (GLA) and glucaric acid (GA) rose from 87% at a pH of 2 to a maximum of 94% at a pH of 7, before decreasing to 78% at pH 10. The flow-through cell (Figure 8b) was believed to limit the expansion of the electrochemical diffusion layer and promote glucose access to anode surface by convection. The effects of current density on glucose conversion to product selectivity were also studied (Figure 8c). With MnO_2_ loading of 4.98%, glucose concentration of 50.5 mM, temperature of 30 °C, pH of 7, residence time of 19 min, and current density of 4 mA/cm^2^, 98% glucose conversion was achieved with selectivity towards gluconic acid and glucaric acid of 43% and 55%, respectively. Increasing current density to 6 mA/cm^2^ further led to 99% glucose conversion, with gluconic and glucaric selectivities of 15% and 84%, respectively. 

In 2017, Solmi et al. investigated the effects of varying ratios of different reactants (glucose to NaOH, glucose to metal catalyst, and glucose concentration levels), temperature and pressure, on glucose conversion to glucaric acid [55]. Under optimal conditions using AuNPs on activated carbon support, the highest yields of glucaric acid, gluconic acid and other by-products attained were 31%, 18% and 40%, respectively. The authors proposed that the optimal glucose concentration was 5% and that the molar ratio of glucose to metal should be 500:1 for obtaining glucaric acid. 

Moggia et al. compared electrooxidative performance of bare copper (Cu), platinum (Pt) and Au at varying potentials for different functional groups [56]. The researchers first compared CVs of 0.04 M glucose added to 0.1 M sodium hydroxide with Cu, Pt and Au electrodes, and concluded that glucose oxidation is strongly correlated with metal catalysts. Next, gluconic acid, glucuronic acid and glucaric acid were used to identify if different functional groups are oxidized by metal catalysts. Cu was found to selectively oxidize glucose aldehyde group at 0.8–1.2 V, but not the hydroxymethyl group. Pt oxidized hydroxymethyl groups at lower potentials and aldehyde groups at higher potentials, while Au oxidized hydroxymethyl at higher potentials and aldehydes at lower ones. Finally, to minimize competing side reactions (glucose isomerization and sugar degradation [57]) the authors performed glucose electroreforming at 5 °C at pH 13 for all three metal catalysts. Although good selectivity to glucaric acid was observed at lower potential (38.4% at 0.86 V), current densities with Cu were too low. Increasing potentials resulted in formic acid as the major product. Pt and Au exhibited similar catalytic action: at low potentials (0.55–0.70 V), both exhibited high selectivity to gluconic acid (78.4–86.8%). Increasing potential to 1.34 V for Pt and prolonged electrolysis at 0.70 V for Au produced glucaric acid selectivities of 13.5% and 12.6%, respectively. 

Moggia and co-workers then went on to optimize conditions for each of the two oxidation steps at Au anodes: (1) glucose to gluconic acid, and (2) gluconic acid to glucaric acid [58]. For the first step, pH, glucose concentration, and temperature were all found to influence conversion. Optimal parameters of pH of 11.3, glucose concentration of 0.04 M, temperature of 5 °C and potential of 0.6 V resulted in the highest gluconic acid selectivity of 97.6%, with glucose conversion of 25% across a 6 h reaction. Increasing conversion by elevating pH or temperature produced lower selectivity. Increasing glucose concentration likely affected mass transfer to Au active sites, which also reduced selectivity. On the other hand, none of the above parameters were significant for oxidizing gluconic acid to glucaric acid. Instead, applied potential considerably influenced the product distribution. As illustrated, the maximum selectivity of 89.5% was attained at 1.1 V vs. RHE. Unfortunately, gluconic acid conversion was low (4.6%). Nevertheless, the highest possible glucaric acid concentration obtained was 1.2 mM. Furthermore, the drastic drop in current density was observed after a few hours, likely due to the adsorption of glucaric acids at Au active sites, as reported previously [59]. 

Liu et al. used both nanostructured bimetallic nickel-iron oxide (NiFeO_x_) and nitride (NiFeN_x_) electrodes for gluconic and glucaric acid production [60]. NiFeO_x_ nickel foam (anode) was used in 0.5 M glucose and 1 M potassium hydroxide, while NiFeN_x_ nickel foam was used as the cathode in 1 M KOH. At a constant applied voltage of 1.4 V, glucose conversion of 21.3% was attained, and glucaric acid and gluconic acid yields were 11.6% and 4.7%, respectively. The Faradaic efficiencies for both glucaric and gluconic acids were 87%. The current density at 1.4 V decreased from 101.2 to 97.8 mA/cm^2^ over a 24 h run. The authors’ technoeconomic analysis suggests that this method produces glucaric acid at 54% lower cost compared to conventional production methods. 

In 2021, Neha et al. created platinum-bismuth alloy (Pt_9_-Bi_1_) electrocatalyst on glassy carbon electrode for glucose conversion to gluconic acid, accompanied by methyl-glucoside conversion to methyl-glucuronate [61]. In an electrolyte consisting of 0.1 M NaOH (0.1 M glucose added), linear sweep voltammetry (LSV) scans showed the onset voltage to be less than 0.06 V and a broad peak at 4.58 mA/cm^2^ around 0.6 to 0.8 V. Chronoamperometric measurement at a fixed potential of 0.3 V was conducted with Pt_9_-Bi_1_/C anode and Pt/C cathode for 6 h, in a filter press cell. After about 90 min, the current halved (~0.020 to 0.010 A) and plateaued at around 0.005 A. These readings suggest that some poisoning occurred. The product after a 6 h reaction was confirmed to be gluconate with 100% selectivity using HPLC and NMR, with 40% glucose conversion. 

**Figure 9:**
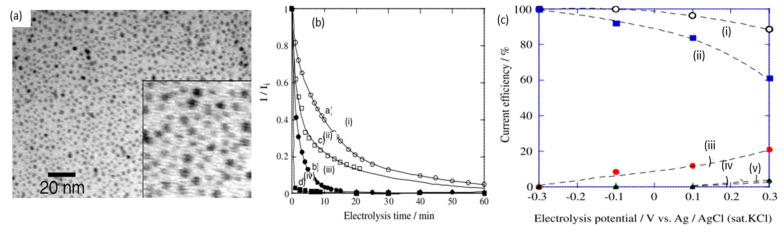
(**a**) TEM image of AuNP capped with decanethiolate monolayer shell (for deposition on electrode). (**b**) Change in current ratios across time with gold nanoparticles in alkali medium (i), bulk gold in alkali medium (ii), gold nanoparticles in neutral medium (iii), and bulk gold in neutral medium (iv). (**c**) Plots of electrolysis products with 2 nm AuNP in 0.1 M NaOH, 0.01 M glucose, at different potentials (vs. Ag/AgCl); total current efficiency of all products (i), gluconolactone (ii), oxalate (iii), glyconate (iv), formate (v). Reprinted with permission from Ref. [59]. Copyright 2005, Electrochemistry Communications.

Poisoning of electrodes is a significant challenge in scaling up glucose electroreforming, suspected to be caused by the action of reaction intermediates [62]. In 2005, Tominaga et al. compared glucose electrooxidation between pure Au plate and AuNPs (2 nm in diameter) on carbon electrode [59], as shown in Figure 9a. While CV scans suggested a similar voltametric response, gold nanoparticle catalysts exhibited significantly smaller decreases in current over time, displaying better resistance to poisoning, as displayed in Figure 9b. The reduction in current density was mitigated by increasing pH. Additionally, Tominaga et al. identified that high selectivity towards gluconate can be obtained at a high pH of 13.7, while electroreforming at neutral conditions (pH of 7) produced a mixture of gluconate and oxalate. Similarly, applied potential can be another factor to influence products, as seen in Figure 9c. Most later studies have therefore focused on employing nanoparticle electrocatalysts in alkali media.

### 2.3. 5-Hydroxylmethylfurfural

5-hydroxylmethylfurfural (5-HMF) was included in a 2010 revision of the US Department of Energy’s list for most valuable chemicals due to its versatility in forming a wide range of useful chemicals. In particular, one of its products, 2,5-furandicarboxylic acid (FDCA), has been widely acknowledged for its potential to replace polyethylene terephthalate (PET) in plastic production with comparable mechanical strength and superior cost savings [63,64,65]. The recent advances in anodic electroreforming of 5-HMF will be discussed in this section.

**Figure 10:**
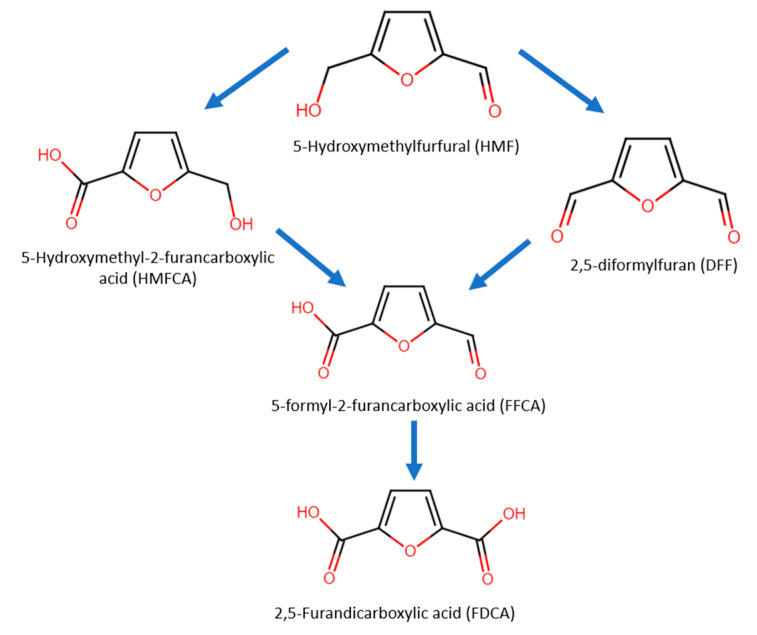
Oxidation pathways and products of 5-HMF.

In 5-HMF oxidation, either the alcohol group or aldehyde group can be oxidized first, forming the intermediates 2,5-diformylfuran (DFF) or 5-hydroxymethyl-2-furancarboxylic acid (HMFCA), respectively (Figure 10). Next, the intermediates further undergo oxidization into 5-formyl-2-furancarboxylic acid (FFCA) and lastly into FDCA. 

In 2018, Liu et al. fabricated NiFe layered double hydroxide (LDH) nanosheets on carbon paper as electrocatalysts to electroreform 5-HMF to 2,5-furandicarboxylic acid (FDCA) [66]. Upon adding 10 mM 5-HMF, LSV scans revealed onset potential at 1.25 V (vs. RHE) occurred earlier than the oxygen evolution reaction (OER) at 1.37 V for double layer capacitance, which further supported the suggestion that there is a greater electrochemically active area for 5-HMF oxidation than that for oxygen evolution. Among the LDH materials, including bimetallic nickel-aluminum (NiAl), nickel-gallium (NiGa), and Ni(OH)_2_, NiFe displayed the best performance. Additional chronoamperometric tests at a potential of 1.33 V resulted in the best 5-HMF conversion of 98% and an FDCA yield of 98%, with Faradaic efficiency of 98.6% in 1 M potassium hydroxide electrolyte with 10 mM 5-HMF. When the concentration of 5-HMF was increased to 100 mM, good conversion, yield, as well as efficiency were still observed (all values >90%). 

**Figure 11:**
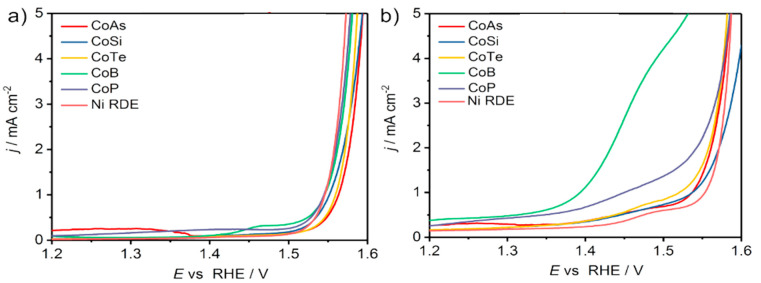
LSV scans of cobalt alloys in half cell in 1 M KOH, (**a**) without the addition of 5-HMF (OER), and (**b**) with the addition of 10 mM 5-HMF (5-HMF oxidation to FDCA). Reprinted with permission from Ref. [67]. Copyright 2018, Beilstein Journal of Organic Chemistry.

Weidner et al. investigated the electrooxidation of 5-HMF to FDCA with bimetallic cobalt-metalloid alloys to replace OER in water splitting [67]. Among cobalt phosphide (CoP), cobalt boride (CoB), cobalt telluride (CoTe), dicobalt silicide (Co_2_Si) and cobalt arsenide (CoAs), CoB had the highest current activity (2.69 mA/cm^2^ at 1.45 V), where OER is negligible, and the lowest onset potential for 1 mA/cm^2^ was 1.39 V, as displayed in Figure 11a,b.

**Figure 12:**
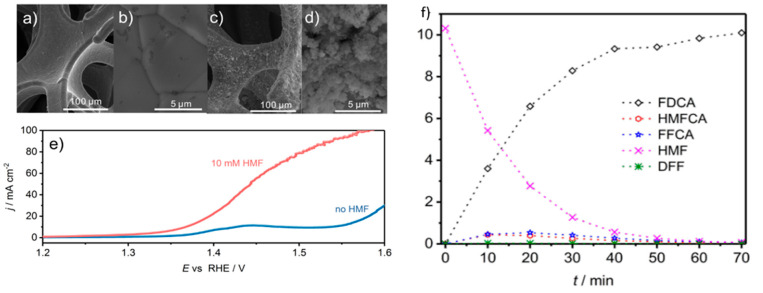
(**a**,**b**) SEM images of nickel foam before CoB deposition. (**c**,**d**) SEM of foam after CoB deposition. (**e**) LSV in flow reactor with and without 5-HMF. (**f**) Concentration against time for various products. Reprinted with permission from Ref. [67]. Copyright 2018, Beilstein Journal of Organic Chemistry.

A flow reactor was then constructed with CoB-doped Ni foam as a positive electrode and Ni foam as a negative electrode, separated by anion exchange membrane. Figure 12 a,b show the smooth surface of the Ni foam, whereas Figure 12c,d reveal the rough appearance of the CoB-doped foam due to agglomerations of CoB after spray coating deposition. As illustrated in Figure 12e, the current density at anodic potential of 1.45 V was 55 mA/cm^2^ with additional 10 mM 5-HMF. Furthermore, by maintaining potential at 1.45 V, complete conversion of 5-HMF was realized. The FDCA yield attained was 94% and Faradaic efficiency was 98%, as seen in Figure 12f. Notably, the degradation of 5-HMF into humin-type structures, typically observed at high pH, was suppressed. Remarkably, the current density at the applied voltage (1.45 V) recorded at 55 mA/cm^2^, much lower than the 1.63 V required for the same current density at OER. 

**Figure 13:**
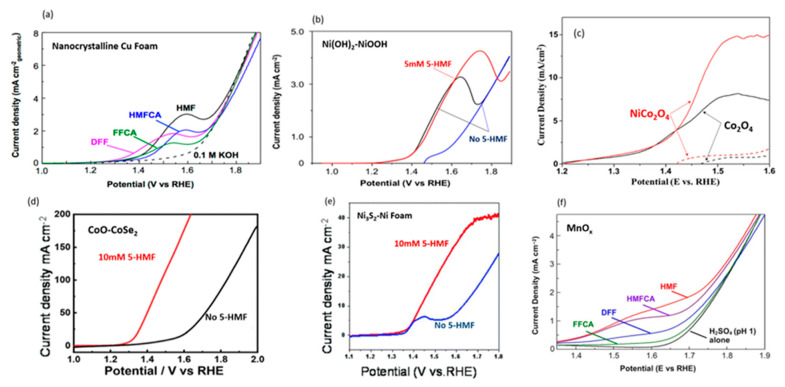
Comparison of LSV graphs with different catalysts. (**a**) LSV scans of 5 mM of 5-HMF and intermediates (solid lines) and without 5-HMF (dashed line) at a pH of 13 using nanocrystalline Cu foam. Reprinted with permission from Ref. [68]. Copyright 2018, ACS Catalysis. (**b**) LSV of Ni(OH)_2_ catalysts without (blue and black lines) and with 5 mM 5-HMF (red line) at a pH of 13. Reprinted with permission from Ref. [69]. Copyright 2019, ACS Catalysis. (**c**) LSV of NiCo_2_O_4_ and Co_3_O_4_ catalysts without (dashed lines) and with (solid lines) 5 mM 5-HMF, at a pH of 13. Reprinted with permission from Ref. [70]. Copyright 2019, Applied Catalysis B: Environmental. (**d**) LSV of CoO-CoSe_2_ electrocatalysts without (black line) and with (red line) 10 mM 5-HMF at a pH of 13. Reprinted with permission from Ref. [72]. Copyright 2020, Green Chemistry. (**e**) LSV of Ni_3_S_2_-Nickel foam without (black line) and with (red line) 10 mM 5-HMF at a pH of 13. Reprinted with permission from Ref. [74]. Copyright 2021, Dalton Transactions. (**f**) LSV of 20 mM 5-HMF and intermediates (colored lines) and without (black line) with MnO_x_ at a pH of 1. Reprinted with permission from Ref. [75]. Copyright 2018, ChemSusChem.

Suspecting that highly alkali conditions favor the degradation of 5-HMF into insoluble humin products, Nam et al. used an electrolyte with a pH of 13 (0.1 M KOH), rather than a pH of 14, for electroreforming 5-HMF to FDCA [68]. Nanocrystalline Cu foam was used as the electrocatalyst due to Cu’s high overpotential requirement for the competing reaction (i.e., oxygen evolution). LSV scans of 5-HMF and intermediates confirmed that complete conversion 5-HMF to FDCA can be accomplished without oxygen evolution, as shown in Figure 13a. Anodic potential of 1.62 V with 0.1 mM 5-HMF at pH of 13 led to 99.9% of 5-HMF conversion, 96.4% of FDCA yield, and 95.3% of Faradaic efficiency. The authors also tested bulk Cu electrode, while maintaining high conversion (99.1%), achieving lower FDCA yield (80.8%) and Faradaic efficiency (79.9%). Although LSV scans indicated lower activity for bulk Cu, with a potential (at 1 mA/cm^2^) of 1.58 V compared to 1.45 V for nanoparticle Cu, it was still significantly lower than the 1.8 V required for OER. From the product analysis, substantial amounts of FFCA were found, suggesting that oxidation of FFCA to FDCA was the rate limiting step. 

In 2019, Taitt et al. went on to compare performances of various transition metal oxyhydroxides such as NiOOH, CoOOH and FeOOH for the oxidation of 5-HMF into FDCA [69]. 5-HMF oxidation was induced at the lowest potential using CoOOH. However, the authors reported that the current density obtained was too low for practical use, and increasing potentials further resulted in OER. On the other hand, FeOOH exhibited no catalytic activity at potentials below OER. Among those tested in 0.1 M KOH with 5 mM 5-HMF, NiOOH appeared to be the best catalyst, attaining 99.8% conversion, 96% yield and 96% Faradaic efficiency at 1.47 V (Figure 13b). These similar results were also obtained previously by Liu’s and coworkers [66].

Kang et al. then investigated the activities of Co-based oxides as electrocatalysts [70]. Specifically, nickel cobaltite (NiCo_2_O_4_) and cobalt (II, III) oxide (Co_3_O_4_) were deposited onto Ni foam. It can be observed from Figure 13c that the OER onset potentials for NiCo_2_O_4_ and Co_3_O_4_ with 1 M KOH were 1.47 and 1.42 V, respectively. However, both dropped to ~1.2 V after adding 5-HMF. NiCo_2_O_4_′s lower Tafel slope indicated the ease of promoting activity, which was confirmed through LSV scans using intermediates (HMFCA and FFCA). At the anodic potential of 1.5 V with 1 M KOH with 5 mM 5-HMF, the conversion and selectivity of 5-HMF to FDCA were 99.6% and 90.8%, respectively. It is noted that a higher portion of Co^3+^ was reduced to Co^2+^ in NiCo_2_O_4_, which accounted for its more superior performance.

In 2020, Cai et al. investigated HMF oxidation to FDCA using nickel (II)-modified covalent-organic framework (COF) film TpBpy-Ni@FTO [71]. However, LSV scan with 0.1 M LiClO_4_ (pH of 13) and 0.5 M 5-HMF revealed that the potential was about 1.7 V for 0.1 mA/cm^2^. Moreover, the maximum yields were relatively low; with an anodic potential of 1.55 V, 0.5 mM 5-HMF resulted in 96% conversion, a 34% yield of intermediate FFCA, and a 58% yield of FDCA.

Huang et al. introduced oxygen vacancies by doping CoO with selenium (Se) to form CoO-CoSe_2_ electrocatalyst with Co:Se molar ratio of 23:1 [72]. The catalysts were dispersed onto carbon paper support, and electrochemical activities investigated through LSV in 1 M KOH, with and without 10 mM 5-HMF. It can be observed from Figure 13d that the onset potential without 5-HMF was reported to be 1.5 V and with 10 mM 5-HMF was 1.3 V. Overall, CoO-CoSe_2_ showed better performance than CoO and CoSe_2_. In a three-electrode cell, anodic potential of 1.43 V fully converted 5-HMF to FDCA with a yield of 99% and Faradaic efficiency of 97.9%. After five cycles, no decrease in yields and conversions were observed. Likewise, a similar setup with a carbon paper-only electrode (without CoO-CoSe_2_) was performed as a reference and showed significantly lower 5-HMF conversion of 58.3%, with trace FDCA (0.5%) and significant humin-type products. 

In 2021, Hu et al. prepared tungsten trioxide (WO_3_) on Ni foam at relatively low temperatures (180 °C) using varying amounts of polyethylene glycol (PEG) additive [73]. Without 5-HMF, onset potential and potential at 20 mA/cm^2^ were 1.32 and 1.6 V, respectively, while the addition of 5-HMF reduced them to 1.18 and 1.44 V, respectively. Conducting CVs at varying scan rates revealed that WO_3_/Ni_0.18_ (0.18 g of PEG per cm^2^ of electrode) had the largest electrochemically active surface area. In a 3-electrode electrochemical cell with 1 M potassium hydroxide and 5 mM 5-HMF at anodic 1.57 V, WO_3_/Ni_0.18_ resulted in conversion of 88.6%, an FDCA yield of 81.5% and a Faradaic efficiency of 79.5%.

Wang et al. performed sulfidation of Ni foam at 120 °C under hydrothermal conditions to produce nickel subsulfide (Ni_3_S_2_) on Ni foam [74]. Further investigations revealed the presence of Ni^2+^ and Ni^0^ species. In 1 M KOH, water oxidation occurred at 1.63 V with 10 mA/cm^2^. Upon the addition of 10 mM 5-HMF, the required potential fell to 1.43 V, as displayed in Figure 13e. In a three-electrode configuration with a graphite counter electrode, maintained at 1.498 V, conversion of 100%, an FDCA yield of 98.3% and Faradaic efficiency of 93.5% were obtained. A control experiment with only Ni foam was conducted to reveal low yield of FDCA (52%), which supports the enhanced electrocatalytic properties of Ni_3_S_2_. 

In contrast to conventional alkali media, Kubota and Choi investigated the oxidation of 5-HMF to FDCA at a pH of 1 [75]. The authors aimed to induce FDCA precipitation at low pH (<2–3), allowing for easier extraction. Using a manganese oxide (MnO_x_) anode, LSVs were performed in 0.05 M sulfuric acid (H_2_SO_4_), with and without 20 mM 5-HMF and intermediates, as shown in Figure 13f. This confirmed the oxidation of 5-HMF oxidation was favored over OER. Notably, 1.49 V was needed for 1 mA/cm^2^ for 5-HMF oxidation. As a control, Pt was scanned under the same conditions, and was found to exhibit insignificant electrolytic activity. In a three-electrode cell setup, the anodic potential of 1.6 V and temperature of 60 °C were maintained. Elevated temperatures improved the kinetics and solubility of FDCA, which limited precipitation of FDCA on anode. After the reaction, the temperature was lowered to precipitate FDCA, as well as intermediate 5-formyl-2-furancarboxylic acid (FFCA). Almost all 5-HMF were converted (up to 99.9%), while it yielded 53.8% of FDCA and the Faradaic efficiency was 33.8%. Apart from FDCA intermediates, maleic acid was also measured with a yield of 21.9%.

### 2.4. Other Biomass Derivatives

Minor research efforts have been devoted to other cellulose-derived biomass such as levulinic acid (C_5_H_8_O_3_). In 2015, Dos Santos et al. performed the oxidation of levulinic acid to 2,7-octanedione with Pt anode in aqueous solution or methanol at pH of 5.5 [76]. Methanol resulted in much a higher Faradaic efficiency (86% vs. 5%) and higher selectivity (47% vs. 27%) than water-based electrolytes, while using water allowed for slightly higher conversions (74% vs. 60%). 4-hydroxy-2-butanone was also produced directly, using Pt at 6 V in 0.2 M NaOH, resulting in 6% selectivity and 5% Faradaic efficiency. The major oxidation product (~45% selectivity) was identified as 3-buten-2-one. Further electrochemical hydrogenation and oxidation of products were also investigated. For instance,1,3-butanediol and 1-butanol were obtained from 4-hydroxy-2-butanone, and octane from valeric acid, as mentioned in [77]. Cathodic electroreforming of levulinic acid will be further discussed in later sections.

Glycerol (C_3_H_8_O_3_) is a by-product triol obtained through the production of biofuel [78]. The growing popularity of biofuel synthesis in the last decade has resulted in a larger supply of glycerol, spurring extensive research into glycerol electroreforming, which is summarized in the reviews [79,80,81]. Most research employs precious metal electrocatalysts for glycerol electroreforming to valuable chemicals. However, in 2019, Liu et al. successfully used CuO to synthesize 1,3-dihydroxyacetone (DHA), a commercially valuable chemical with applications in cosmetics and polymer industries [82,83]. At the applied current density (3 mA/cm^2^) and pH level 9, a selectivity of 60% to DHA was achieved. 

**Figure 14:**
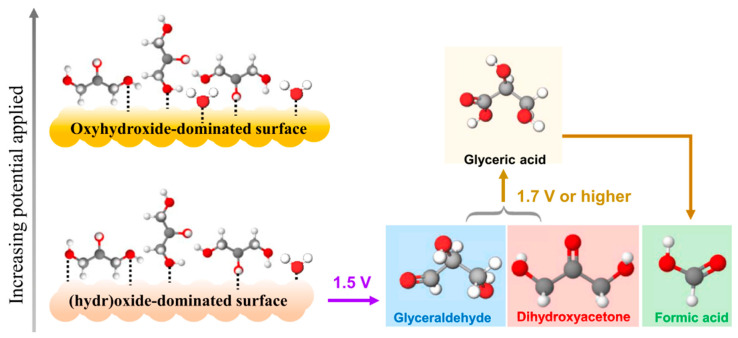
Proposed glycerol oxidation pathway over CoO_x_ in mild alkali media. Reprinted with permission from Ref. [84]. Copyright 2021, Applied Catalysis B: Environmental.

In 2021, Vo et al. utilized CoO_x_ catalysts for anodic glycerol valorization at a pH of 9 [84]. Operando characterization was performed to track the surface species of electrocatalysts, and oxidation pathways were proposed, as shown in Figure 14. CoO_x_ was first electrochemically oxidized into oxyhydroxides to form active sites. Then, glycerol underwent indirect electron transfer to be incompletely oxidized to DHA or glyceraldehyde, or completely oxidized to formic acid. Spectroscopy results indicated that incomplete oxidation was more likely to occur, although formic acid was present at all applied potentials. At an applied potential of 1.5 V, a DHA selectivity of 60% was attained. 

Sorbitol (C_6_H_14_O_6_) is a biomass-derived polyol identified as a promising platform chemical [46]. While most electrochemical studies on sorbitol focused on anodic oxidation in fuel cells, in 2019, Kwon et al. electrochemically oxidized sorbitol to glucose, gulose, fructose and sorbose on a Sb-modified Pt anode in a Bi-saturated pH 3 solution [85]. Although high selectivity to a single product was not obtained, selectivity to value-added products was shown to be influenced by potential. The researchers suggest that more studies might uncover a new pathway to electrochemically convert glucose to fructose through sorbitol.

Furfural (C_5_H_4_O_2_) is one of the oldest biomass-derived chemicals which can undergo electrochemical hydrogenation to form 5-HMF [86,87]. Recent works have also demonstrated anodic electroreforming of furfural to maleic acid, which is widely used in the synthesis of resins and pharmaceuticals. In 2018, Kubota and Choi used PbO_2_ electrodes in acidic media with a pH of 1 and found that the onset current decreased from 1.85 to 1.6 V upon the addition of 10 mM furfural [88]. Furfural was first oxidized to 2-furanol and finally to maleic acid with a yield of 65.1%. In 2020, Roman et al. also reported anodic reforming of furfural to furoic acid, a chemical to produce 2,5-furandicarboxylic acid [89]. A Faradaic efficiency of 96% was attained for furoic acid on Au electrodes at 0.8 V and a pH of 0.6, although low current densities (<30μA/cm^2^) suggest the rate of reaction might be slower than desired. Notably, through density functional theory and attenuated total reflectance surface-enhanced infrared absorption spectroscopy (ATR-SEIRAS) studies, the desorption of furoate from electrode surface was suggested as the rate-limiting step at Au and Pt electrodes. This hypothesis was validated using further CV characterizations of furfural oxidation in the presence and absence of furoic acid. Small quantities (1%) of furoic acid were sufficient to inhibit furfural oxidation in acidic conditions.

Studies on electroreforming of cellulose and its derivatives as well as other biomass derivatives at the anode are summarized in Table 1 with the key technical information.

## 3. Electroreforming of Biomass at the Cathode

In addition to oxidation at the anode, hydrogenation or reduction can also be conducted at the cathode of electrolytic cells. In hydrogenation, H^+^ ions in the solution are reduced to surface-bound atomic hydrogen. Oxygenated organic molecules can react with adsorbed hydrogen to form valuable products. This section details some case studies of electroreforming biomass-derived compounds via cathodic reactions. 

### 3.1. Cellulose

**Figure 15:**
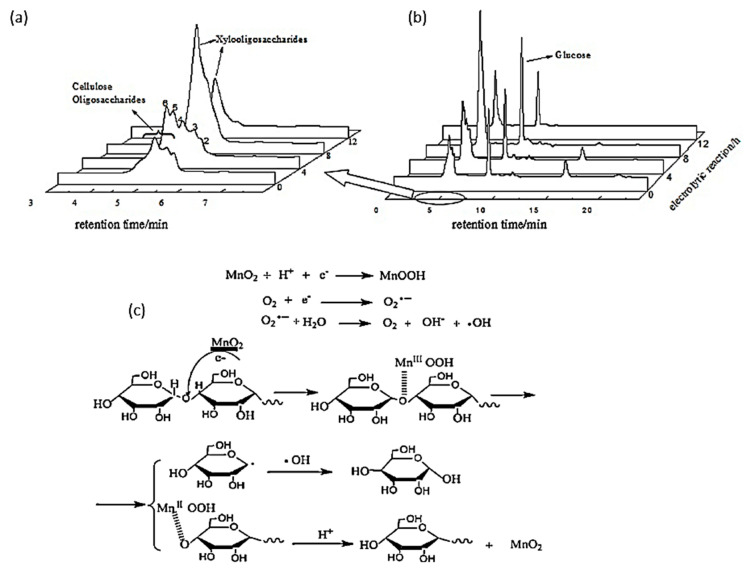
(**a**) HPLC of oligosaccharides after electrolysis. (**b**) HPLC of products after hydrothermal treatment of cellulose. (**c**) Proposed mechanism for cellulose oligosaccharide depolymerization with MnO_2_ cathode. Reprinted with permission from Ref. [90]. Copyright 2014, Journal of Industrial and Engineering Chemistry.

In 2014, Yang et al. investigated the electroreduction of cellulose oligosaccharides into glucose in acidic media [90]. Short chain oligosaccharides were first produced via hydrothermal treatment with an acidic catalyst. The authors varied the pH, applied voltage, electrolysis duration, and electrode preparation to optimize glucose yield. With a 5% MnO_2_/graphite/polytetrafluoroethylene (PTFE) cathode calcinated at 500 °C for 3 h, a glucose yield of 72.4% with 100% selectivity was reported under optimal electrolysis conditions (pH of 3, 8-h reaction duration, and potential of −0.58 V vs. RHE). Figure 15a,b depicted the product analysis by HPLC before and after electroreforming.

Similar to the electrooxidation mechanism with a gold electrode proposed by Sugano’s group [35], Yang et al. hypothesized that cellulose would first adsorb onto the surface of a MnO_2_/graphite/PTFE cathode, as shown in Figure 15c. Mn (VI) (in MnO_2_) would be reduced to Mn (III) (in MnOOH) after reaction with a H^+^ ion and electron. Afterwards, MnOOH coordinates with oxygen in the glycosidic bond, depolymerizing the cellulose chain. Lastly, MnOOH is re-oxidized into MnO_2_ in the acidic medium.

### 3.2. 5-Hydroxylmethylfurfural

**Figure 16:**
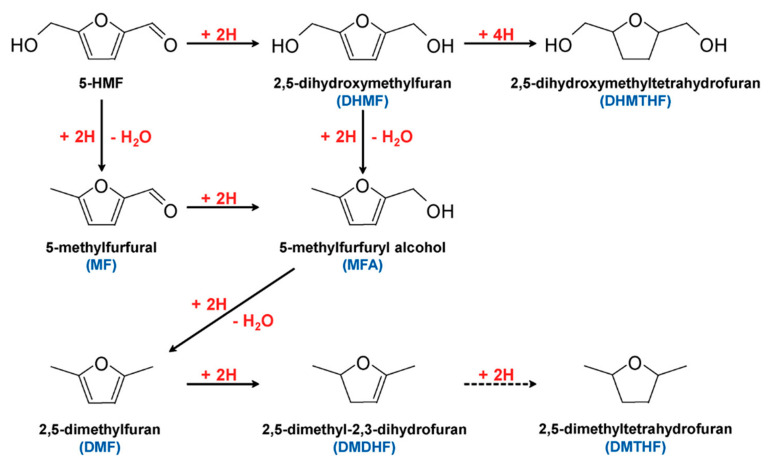
Hydrogenation pathways of 5-HMF. Reprinted with permission from Ref. [92]. Copyright 2015, ChemSusChem.

5-HMF can undergo hydrogenation to form valued products, as shown in Figure 16. Products such as 2,5-dimethylfuran (DMF) are considered potential gasoline alternatives, due to their high carbon and energy density [66,91,92,93]. 2,5-dihydroxymethylfuran (DHMF) is a platform chemical for polyester and polyurethane foam production [94]. Conventional hydrogenation of 5-HMF often requires high temperatures and/or pressures, as well as hydrogen atmosphere [95]. Electrochemical hydrogenation represents an attractive alternative to produce these chemicals without requiring harsh conditions.

In 2013, Nilges and Schroder first demonstrated electrochemical hydrogenation of 5-HMF to DMF [87]. At a constant 10 mA/cm^2^ in a 0.5 M sulfuric acid electrolyte with Cu electrodes, the highest DMF selectivity of 35.6% was attained. As reaction proceeded, a significant decrease in Faradaic efficiency was observed alongside a decrease in 5-HMF concentration in the solution. The authors proposed that a flow reactor would allow for sparingly soluble DMF products to be continuously removed, therefore maintaining high Faradaic efficiencies for 5-HMF electroreforming. 

Kwon et al. explored electrocatalytic hydrogenation of 5-HMF using different metal catalysts in neutral media (0.1 M sodium sulfate buffer, pH ~7.2) in the presence and absence of glucose [91]. Based on the similar onset potentials (~−0.5 V) observed for all metals, they found that the rate of electrocatalytic hydrogenation is not strongly influenced by the catalyst. However, the choice of catalyst was found to influence hydrogenation pathway and products. Broadly, the metal catalysts used were classified into three groups depending on products obtained in neutral media. Firstly, Fe, Ni, silver (Ag), zinc (Zn), cadmium (Cd), and indium (In) formed DHMF as the major product. Secondly, hydrogenolysis products of 5-HMF were mainly formed on Co, Au, Cu, tin (Sn), and antimony (Sb). Finally, using palladium (Pd), Al, bismuth (Bi), and Pb could form either DHMF or other hydrogenolysis products by controlling the applied potential. Upon the addition of glucose, Zn, Cd, In, Fe, Ni, Ag, Co, and Au electrocatalysts favored the hydrogenation of 5-HMF into DHMF, while no such effects of initial glucose concentration on final product preference were observed using Bi, Pb, Sn or Sb. 

Similarly, different metal electrocatalysts were tested in acidic media (0.5 M H_2_SO_4_, pH of 0.3) [92], and classified into three groups according to major products. In acidic media, Fe, Ni, Cu, and Pb formed DHMF as the major product. 2,5-dimethyl- 2,3 dihydrofuran (DMDHF) was the major product using Pd, Pt, Al, Zn, In, and Sb. Using Co, Ag, Au, Cd, Sb, and Bi formed either DHMF or DMDHF as the major product by controlling the applied potential. 

In 2019, Chadderdon et al. performed the paired electrocatalytic hydrogenation of 5-HMF at the cathode to produce 2,5-bis(hydroxymethyl)furan (BHMF) [96]. The hydrogenation reaction was catalyzed by Ag nanoparticles on carbon support in 0.5 M borate buffer solution of pH 9.2. Electrooxidation of 5-HMF at the anode was performed with homogenous 4-acetamido-TEMPO catalysts. Pairing these anodic and cathodic electroreforming reactions achieved co-generation of valuable products.

**Figure 17:**
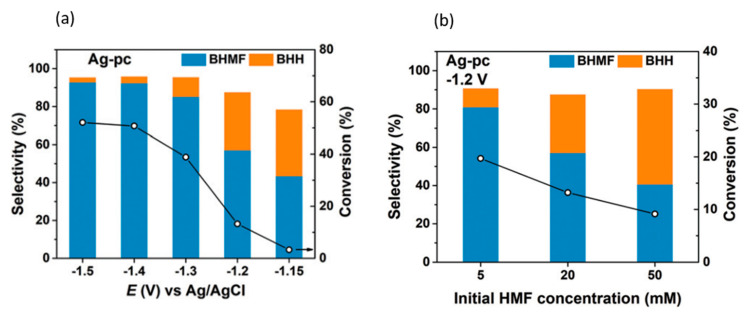
(**a**) Product selectivities at different cathodic potentials (vs. Ag/AgCl). (**b**) Product selectivities at different 5-HMF concentrations. Reprinted with permission from Ref. [96]. Copyright 2019, Green Chemistry.

5-HMF hydrogenation products were found to be dependent on cathodic potential and 5-HMF concentration, as shown in Figure 17a,b. Increasing the potential or 5-HMF concentration shifted selectivity towards 5,5-bis(hydroxymethyl)hydro-furoin (BHH) rather than BHMF. At the optimum conditions of −0.46 V (vs. RHE) and a 5-HMF concentration of 5 mM, 5-HMF conversion reached 19.7%, while the selectivity and Faradaic efficiency of BHMF production were highest, at 80.9% and 89.3%, respectively. 

Zhang et al. investigated acidic media for the electrocatalytic hydrogenation of 5-HMF into DMF [97]. Specifically, a bimetallic CuNi electrode (composed of 82% Cu, 17% Ni and trace O_2_) was synthesized. After that, LSV scans in 0.2 M sulfate buffer (pH of 2) were performed. The scans showed a considerable decrease in the magnitude of potential required for 5 mA/cm^2^ when 2 g/L (15.9 mM) 5-HMF was added (−0.6 V vs. −0.36 V), suggesting good electrocatalytic activity for 5-HMF reduction. When the anodic potential of −0.46 V (vs. RHE) was maintained for 70 s, a maximum selectivity to DMF of 91.1% and a Faradaic efficiency of 84.6% were obtained. Under these conditions, however, the conversion of 5-HMF was low, at 37.8%.

**Figure 18:**
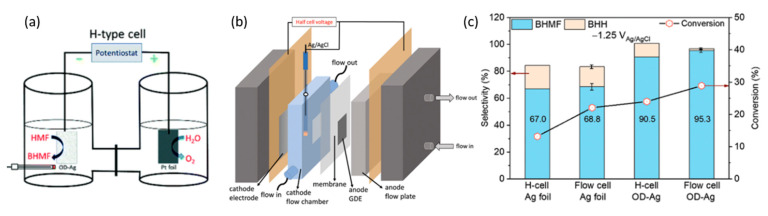
(**a**) H cell diagram. (**b**) Flow cell diagram. (**c**) BMHF and 5,5-bis(hydroxymethyl)hydrofuroin (BHH) results from 5-HMF hydrogenation using Ag or OD-Ag, in H cell or flow cell. Reprinted with permission from Ref. [98]. Copyright 2021, Green Chemistry.

In 2021, Liu et al. conducted electrocatalytic hydrogenation of 5-HMF with Ag foil and oxide-derived Ag (OD-Ag) electrodes [98]. These reactions occurred in 0.5 M borate buffer at pH of 9.2 and 20 mM of 5-HMF. Figure 18a,b demonstrates the electrolysis setup that was performed in an H-cell and the-electrode flow cell, respectively. At cathodic potential of −0.51 V (vs. RHE), 5-HMF conversion and selectivity to 2,5-bis(hydroxymethyl)furan (BHMF) were higher with OD-Ag electrocatalysts instead of Ag foil, and in the flow cell rather than the H-configuration cell. Consequently, this led to the highest 5-HMF conversion of almost 30%, and a selectivity to BHMF of 95.3% (Figure 18c).

Likewise, using OD-Ag in a flow cell, cathodic hydrogenation of 5-HMF into BHMF was coupled with TEMPO electromediated oxidation of 5-HMF into FDCA at a platinum anode. Overall, the cell energy efficiency of the flow cell was higher than the H-cell, i.e., 24.5% compared to 5.7%. The resultant selectivity to BHMF was consistently around 90%, accompanied by complete selectivity to FDCA.

### 3.3. Levulinic Acid

**Figure 19:**
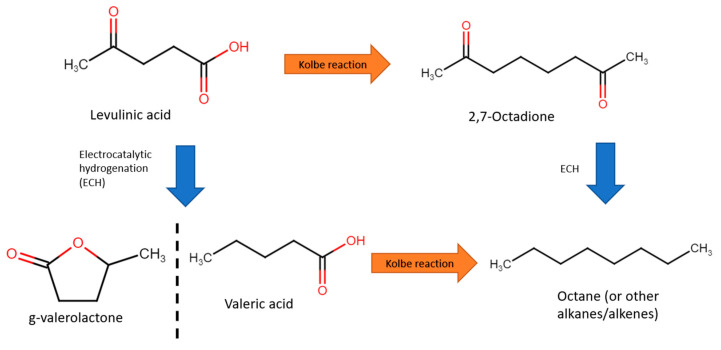
Reaction pathways from levulinic acid to octane.

Combustion of biofuels could result in zero net carbon release into the atmosphere, representing a greener mode of energy production [99,100]. For instance, one example of a biofuel is octane, which can be produced from levulinic acid. The traditional reforming of levulinic acids calls for elevated temperatures and pressures (250–400 °C and 10–35 bar), which require significant energy resources to sustain [101,102,103]. Electroreforming levulinic acid might be an attractive alternative for synthesising hydrocarbons for energy generation. This is usually performed at the cathode (reduction) under acidic media, and consists of two steps: the Kolbe reaction and electrocatalytic hydrogenation (ECH), as shown in Figure 19. Several such studies will be explored in this section.

In 2012, Nilges et al. first performed ECH of levulinic acid to valeric acid with lead cathode [77]. Valeric acid was then converted to octane via the Kolbe reaction with a Pt cathode. Intially, ECH was performed in 0.5 M sulfuric acid (pH of 1) and 0.1 M levulinic acid at a fixed −1.405 V vs. RHE, with a current density of 20–40 mA/cm^2^. With a Pb electrode, Faradaic efficiency of 27% and selectivity to valeric acid of 97.2% were achieved. Subsequently, for the Kolbe step, water and methanol as solvents were compared. Overall, water resulted in better activity, with 40–50 mA/cm^2^ at 3.895 V, achieving selectivity of 51.6% and Faradaic efficiency of 66.5%. In addition, easier extraction of water insoluble products, of which, at 1 M valeric acid and pH of 5.5, 72% octane selectivity was achieved.

In 2013, Xin et al. studied the electroreforming of levulinic acid to valeric acid and g-valerolactone [104]. Identical to valeric acid, g-valerolactone is an essential precursor to biofuel [105] or can be blended into gasoline directly [106]. The authors compared CVs of Cu and Pb electrodes at pH of 0, and found that the onset potential of Cu was of lower magnitude than that of Pb (−0.4 V vs. −1.1 V). However, upon adding 0.2 M levulinic acid, onset potential of Pb increased by 0.2 V while Cu displayed little change, suggesting that adsorption of levulinic acid on Cu was suppressed by fast hydrogen evolution reaction (HER). At low overpotentials (−1.1 V) with a Pb electrode, conversion of 1.2% and selectivities towards valeric acid and g-valerolactone of 81.5% and 18.5%, respectively, were attained. In contrast, higher overpotentials (−1.5 V) led to conversions of 20.3% with selectivity of 97% to valeric acid. Additionally, the effects of pH were studied by contrasting CVs in 0.5 M sulfuric acid (pH of 0) and phosphate buffer (pH of 7.5). In an acidic medium, this resulted in 94% selectivity to valeric acid with 12.7% conversion and 84% Faradaic efficiency. The opposite behavior in neutral medium was observed with 100% selectivity to g-valerolactone, although conversion and Faradaic efficiency were low (1.3% and 6.2%, respectively).

**Figure 20:**
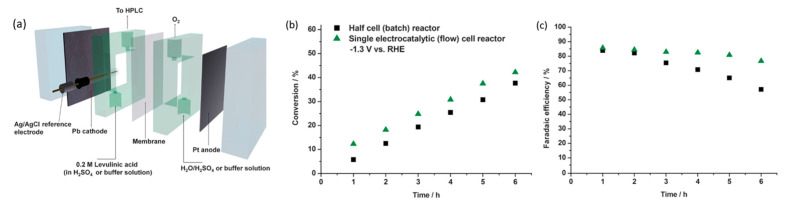
(**a**) Schematic of flow cell reactor. (**b**) Comparison of conversion and (**c**) Faradaic efficiencies of half-cell against flow cell reactor, at −1.3 V, 0.2 M levulinic acid, 0.5 M H_2_SO_4_, with Pb electrode. Reprinted with permission from Ref. [104]. Copyright 2013, ChemSusChem.

Xin’s and coworkers then constructed a flow cell for continual electrolysis with applied potential fixed at −1.3 V (Figure 20a). When operating in the flow cell reactor, higher efficiencies and conversion were recorded accordingly in Figure 20b,c. This was likely due to the high flow rate, which addressed the mass transport issues. Stability tests were conducted across a 20 h reaction with 0.2 M levulinic acid. Conversion rates were consistent and no Pb leaching was observed, although the efficiency of Faradaic processes decreased over time. 

In 2015, Dos Santos et al. performed numerous electroreductions of levulinic acid with various electrode materials (C, Cu, Fe, Ni, Pb) and pH (acid, alkali or neutral), and compared their products and yields [76]. All ECH was performed at cathodic potentials of −1.8 V. At a pH of 0, Pb resulted in the best yield of valeric acid, with over 70% conversion and 80% selectivity to valeric acid. This is likely due to Pb’s high overpotential for hydrogen evolution, which is the competing reaction at the cathode. On the other hand, using carbon in acidic conditions, or Fe in alkali conditions (pH of 14) results in g-valerolactone as the major product, with 40% conversion and selectivities around 70%. 

In 2020, Du et al. compared different electrocatalyst materials (Pt, Pb, Zn, Ti, Co and Cu) for ECH of levulinic acid to valeric acid [107]. Overall, Pb showed the best balance of conversion, selectivity, and Faradaic efficiency. Generally, decreasing the applied potential led to higher conversion and selectivity but lower Faradaic efficiency. While reducing pH led to higher conversion of levulinic acid, it also caused the Faradaic efficiency to decline. As more hydrogen adsorbed on the surface, hydrogen gas formation (via Tafel step or Heyrovsky step) was enhanced. Further LSV scans with different concentration of levulinic acid (0 to 0.5 M) in 0.5 M sulfuric acid showed ECH of levulinic acid was favored over hydrogen evolution with levulinic acid concentrations above 0.1 M. This led to a potential of −1.15 V at a current density of −0.1 mA/cm^2^. With elevated temperatures, the overall conversion and rate were increased, and the authors concluded that optimal conditions for ECH of levulinic acid to valeric acid with Pb cathode were 0.5 M sulfuric acid with 0.2 M levulinic acid, at −1.60 V and 65 °C over 4 h. Additional stability tests were conducted at room temperature with the mentioned optimal condition, across eight 4-h cycles. Consequently, the selectivity towards valeric acid, Faradaic efficiency, and conversion remained high at 90%, 94% and 48%, respectively. 

To support the development of electroreforming on a larger scale, Kurig et al. studied the production of 2,7-octanedione from levulinic acid in a continuous flow single pass reactor [108]. Their setup involved Pt electrodes in 1 M levulinic acid and 0.1 M KOH as the supporting electrolyte. The optimal residence time (volume of reactor/flow rate) was found to be 36 min, resulting in levulinic acid conversion of 48% and selectivity of 52%. Unfortunately, even under optimal conditions, the performance of the single pass reactor was poorer than that using a semi batch cell, which could achieve 100% conversion and 75% yield. The authors proposed that, for Kolbe electrolysis, localization of radicals was important to promote decarboxylation. 

Studies on electroreforming of cellulose and its derivatives at the cathode are summarized in Table 2 with the key technical information.

## 4. Evolution of Hydrogen Coupled with Biomass Electroreforming

Owing to the ever-increasing demand for green hydrogen, the decoupling of HER and OER by replacing OER with biomass oxidation has attracted intensive attention recently. Thus, the generation of byproducts of biomass electrooxidation, i.e., green hydrogen, has been intentionally optimized in addition to the enhancement of biomass electrooxidation.

### 4.1. Glucose Electrooxidation Coupled with Green Hydrogen Generation

**Figure 21:**
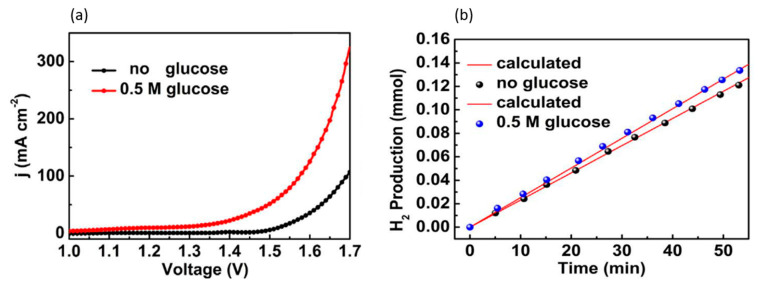
(**a**) LSV scan in 2-electrode cell without and with 0.5 M glucose in 10 M KOH, iron phosphide anode. (**b**) Amount of hydrogen theoretically calculated and collected at 1.9 V, 10 M KOH with and without 0.5 M glucose. Reprinted with permission from Ref. [109]. Copyright 2017, Electrochemistry Communications.

Using glucose electrooxidation instead of OER to generate green hydrogen for the first time, Du et al. presented the results of their research in 2017 [109]. In their work, iron phosphide films were prepared in situ on stainless steel mesh and used as anodes. The anodic potentials (vs. RHE) to operate at the current density of 10 mA/cm^2^ without and with glucose (of 0.5 M concentration) were 1.52 and 1.22 V, respectively, as compared in Figure 21a. No effervescence was observed at the anode, suggesting glucose oxidation completely replaced OER. At a fixed potential of 1.9 V, the hydrogen production rate that coupled to glucose oxidation was higher than that for regular water electrolysis where hydrogen production was coupled to OER, as seen in Figure 21b.

**Figure 22:**
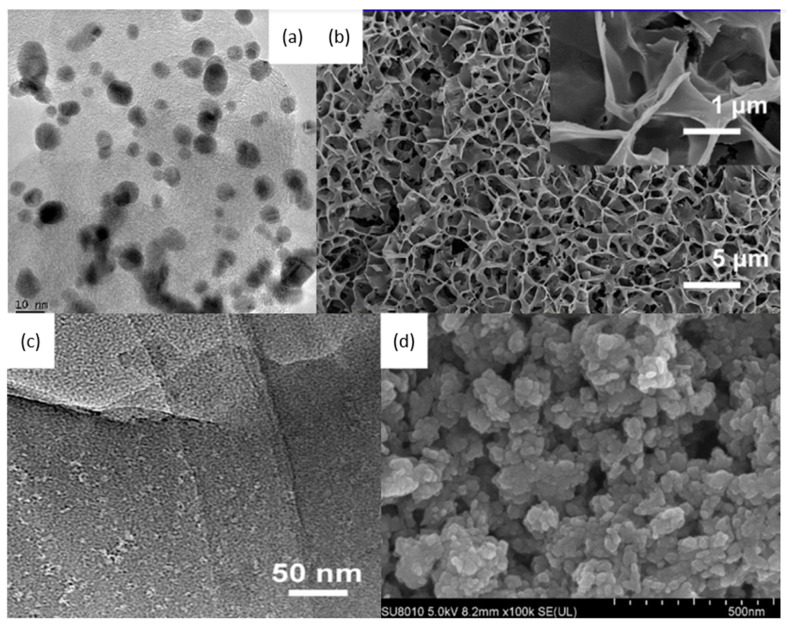
(**a**) TEM of Pd_3_Au_7_/C, reprinted with permission from Ref. [110]. Copyright 2019, Applied Catalysis B: Environmental. (**b**) SEM of Co-Ni alloy, reprinted with permission from Ref. [112]. Copyright 2020, Journal of Alloys and Compounds. (**c**) SEM of Co_0.5_ Ni_0.5_(OH)_2,_ reprinted with permission from Ref. [113]. Copyright 2020, Journal of Electroanalytical Chemistry. (**d**) SEM of Ni-MoS_2,_ reprinted with permission from Ref. [114]. Copyright 2020, International Journal of Hydrogen Energy.

In 2019, Rafaïdeen et al. designed Pd–Au nanoparticles on carbon for glucose and xylose oxidation [110] (Figure 22a). The optimal ratio of Pd to Au composition was found to be 3:7. In 0.1 M NaOH (pH of 13) and 0.1 M glucose, CV revealed the potential required for 1 mA/cm^2^ was 0.2 V, with peak current density (4 mA/cm^2^) at 0.5 V. Notably, the conversion of glucose was 67%, and the selectivity to gluconic acid was 87%. Hydrogen production was observed at Pt/C cathode without further characterization.

Moreover, the effects of glucose concentration and potentials on the reaction were investigated [111]. Increasing glucose concentration beyond 0.1 M was found to result in lower Faradaic efficiency and chemical yields, suggesting surface poisoning. Unsurprisingly, rate of gluconic acid production was higher with higher voltage, although Faradaic efficiency decreased. The production of hydrogen was estimated by calculating charge transfer to form the measured gluconic acid. It was proposed that, theoretically, 1 ton of 0.1 M glucose could produce 18.47 kg of hydrogen at 0.6 V with the setup, consuming 297 kWh electricity. However, it should be noted that this assumes no degradation of electrodes.

In 2020, Lin et al. investigated the application of Co-Ni alloy electrocatalysts on carbon cloth [112] (Figure 22b). SEM imaging showed significant macropore distribution, and X-ray photoelectron spectroscopy characterizations suggested that the partial oxidation of alloy occurred. Additional LSV determined the potential for 10 mA/cm^2^ on Co-Ni alloy electrode to be 1.096 V in 1 M KOH with 0.1 M glucose, which was less than those on the bulk Co (1.143 V) or Ni (1.138 V) electrodes. Further electrochemical impedance spectroscopy (EIS) measurement and Tafel slope comparisons among Co, Ni and Co-Ni alloy electrodes showed the superior conductivity and kinetics of the alloy electrode. In a two-electrode cell with Co-Ni alloy, both electrodes achieved 10 mA/cm^2^ at a voltage of only 1.39 V in 1 M KOH with 0.1 M glucose.

Lin and coworkers also used a customized cobalt nickel hydroxide nanosheet (Co_0.5_ Ni_0.5_(OH)_2_ NS) on carbon cloth as the anode, and replaced OER with glucose oxidation [113] (Figure 22c). Co_0.5_ Ni_0.5_(OH)_2_ NS electrode measured potential of 1.17 V at current density of 10 mA/cm^2^. After a 12 h reaction at constant current density, the potential increased slightly to 1.20 V, indicating superior electrode stability. In a two-electrode cell with Pt cathode, the required potentials for 10 and 100 mA/cm^2^ in 1 M KOH only were 1.47 and 1.75 V, respectively. With the addition of 0.1 M glucose, the corresponding potentials decreased to 1.22 V and 1.56 V, respectively. 

Similarly, Liu et al. fabricated nickel-molybdenum disulfide (Ni-MoS_2_) for both anode and cathode [114] (Figure 22d). LSV showed the potentials needed for 10 mA/cm^2^ in 1 M KOH without and with 0.3 M glucose were 1.64 and 1.46 V, respectively. Additional Tafel slopes, EIS spectra, and double layer capacitance characterizations revealed preferable kinetics and catalytic activity of Ni-MoS_2_ compared to MoS_2_ and Pt/C, as well as good stability through 12 h chronoamperometric tests. Subsequently, a two-electrode cell was constructed with Ni-MoS_2_ on carbon paper as both the anode and cathode, and 1.67 V was required to reach 10 mA/cm^2^. No bubbles were observed at the anode, suggesting complete suppression of OER.

**Figure 23:**
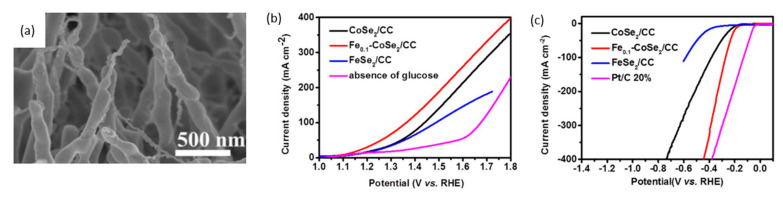
(**a**) SEM image of Fe_0.1_CoSe_2_/CC. (**b**) LSV for anodic glucose oxidation, 1 M KOH, 0.5 M glucose with different electrodes. (**c**) LSV for cathodic hydrogen evolution, 0.5 M H_2_SO_4_. Reprinted with permission from Ref. [115]. Copyright 2020, Applied Catalysts B: Environmental.

Zheng et al. used iron-doped cobalt diselenide nanowires on conductive carbon cloth (Fe_0.1_-CoSe_2_/CC) as an alkaline anode and acidic cathode to produce gluconate (salt of gluconic acid) and hydrogen, respectively [115] (Figure 23a). For a glucose oxidation reaction in 1 M KOH, LSV scans reveal that a Fe_0.1_-CoSe_2_/CC electrode with and without 0.5 M glucose necessitated voltages of 1.65 V and 1.12 V, respectively, as shown in Figure 23b. With glucose, no bubbles were observed at the anode, suggesting complete suppression of OER. Moreover, the chronopotentiometric scans showed stable potential responses, signifying stable mass transport properties. The stability of the electrode was confirmed by XRD and morphological scans taken before and after 8 h of electrolysis at 1.15 V, showing no sign of differences.

The authors also analyzed cathodic hydrogen evolution using Fe_0.1_-CoSe_2_/CC in 0.5 M H_2_SO_4_, and found that overpotential of 270 mV was required to reach a current density of 100 mA/cm^2^ (Figure 23c). Similar tests were conducted to confirm catalytic activity and electrode stability. A two-electrode cell was constructed with a bipolar membrane separating 1 M KOH anolyte and 0.5 M H_2_SO_4_ catholyte. To reach 10 mA/cm^2^, the cell potential in the absence of glucose was applied at 1.34 V, which decreased to 0.72 V with the addition of 0.5 M glucose. For generating green hydrogen, the cell was maintained at 10 mA/cm^2^, with 1 M KOH, 0.5 M glucose at the anode and 0.5 M H_2_SO_4_ at the cathode. Performing chronopotentiometric electrolysis for 100 min yielded 0.15 mmol of H_2_ with 99% Faradaic efficiency.

**Figure 24:**
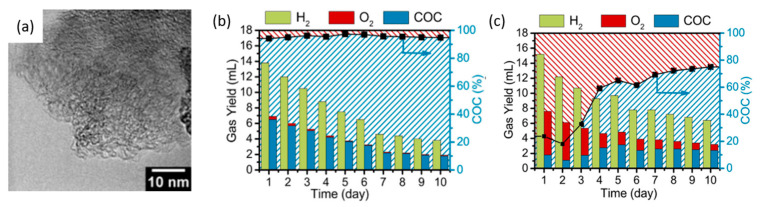
(**a**) SEM image of carbon pellet. (**b**) Hydrogen production over days, from carbon oxidation contribution (COC) or oxygen evolution, with a carbon electrode. (**c**) Hydrogen production under similar conditions with a nitrogen-doped carbon electrode. Reprinted with permission from Ref. [116]. Copyright 2020, ChemSusChem.

Ding et al. further proposed that in order for hydrogen electrolysis to be truly green, electrodes should be part of a closed material cycle [116]. Interestingly, they developed carbon electrodes from biowaste to replace OER with carbon oxidation, intending for the carbon anode to be consumed in the process. Rather than electrolyzing glucose in solution, the authors fabricated carbon pellets (Figure 24a) by means of hydrothermal treatment of glucose, which were deposited onto glassy carbon electrodes. The two-electrode cell potential was fixed at 2.4 V, and the sacrificial carbon anode and Pt cathode were deployed. This anode was maintained at a pH of 13, and the carbon anode was oxidized to carbonate, allowing continuous hydrogen formation at the cathode. The test cell was left to run for 10 days, and the products were quantified (Figure 24b). Doping carbon pellets with nitrogen resulted in better anode stability without influencing electrode conductivity or hydrogen production, as seen in the higher H_2_ evolution after 10 days in Figure 24c compared to that without nitrogen doping in Figure 24b.

### 4.2. 5-HMF Electrooxidation Coupled with Green Hydrogen Generation

5-HMF electrooxidation can also replace OER for safe green hydrogen generation. Yang et al. explored the electroreforming of 5-HMF and simultaneous hydrogen production [117] using Mo-doped nickel selenides on Ni foam (Mo-Ni_0.85_Se/NF). With Mo-Ni_0.85_Se/NF as both electrodes, in 1 M KOH, to achieve current density of 50 mA/cm^2^, adding 10 mM 5-HMF reduced the overall potential from 1.68 to 1.50 V. At an anodic potential of 1.4 V, complete conversion was observed, and Faradaic efficiency and selectivity were both high at >95%, while 3.8 mmol of hydrogen was produced with a Faradaic efficiency close to 100% at the cathode. The authors proposed Mo doping changed the d-band centre of Ni and reduced the hydrogen adsorption energy on electrode surface, increasing electrocatalytic activity. 

**Figure 25:**
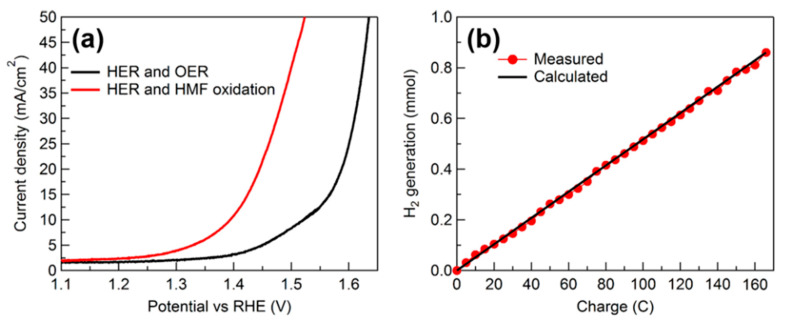
(**a**) LSV of two-electrode cell for concurrent 5-HMF oxidation and hydrogen evolution. (**b**) Amount of hydrogen theoretically calculated and measured at the cathode. Reprinted with permission from Ref. [118]. Copyright 2016, ACS Energy Letters.

Jiang et al. used Co-P catalysts on Cu foam for concurrent anodic oxidation of 5-HMF into FDCA and cathodic reduction of water to H_2_ [118]. In 1 M KOH, anodic potential required for current density of 20 mA/cm^2^ decreased from 1.53 to 1.38 V upon addition of 50 mM 5-HMF. From periodic HPLC analysis during anodic reaction process, suggesting that FDCA is obtained through DFF route (see Figure 10 for reaction pathways). The authors then employed a two-electrode cell for concurrent anodic 5-HMF oxidation and cathodic hydrogen evolution. In 1 M KOH and using Co-P/Cu foam electrodes, the overall potential required for 20 mA/cm^2^ decreased from 1.59 to 1.44 V after adding 50 mM 5-HMF (Figure 25a). In this case, 100% 5-HMF conversion and 90% FDCA yield were measured at the anode, while 8 mmol of hydrogen gas was produced with 100% Faradaic efficiency at the cathode, as depicted in Figure 25b. 

**Figure 26:**
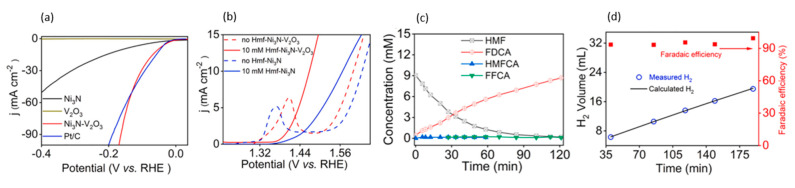
(**a**) LSV for Ni_3_N-V_2_O_3,_ Ni_3_N, V_2_O_3_ and Pt/C cathodes. (**b**) LSV for Ni_3_N-V_2_O_3_ and Ni_3_N anodes with and without 10 mM 5-HMF. (**c**) Concentrations of 5-HMF and products over time, in two-electrode test cell, 1 M KOH, 10 mM 5-HMF, chronopotentiometric test at 10 mA/cm^2^, Ni_3_N-V_2_O_3_ anode and cathode. (**d**) Theoretical and actual production of hydrogen gas. Reprinted with permission from Ref. [120]. Copyright 2021, Chemical Engineering Journal.

Combining Ni and vanadium oxides was found to enhance charge redistribution and weaken hydrogen adsorption on Ni, which would otherwise limit catalytic activity [119]. Thus, Liang et al. fabricated a nickel nitride-vanadium trioxide (Ni_3_N-V_2_O_3_) catalyst for 5-HMF oxidation and hydrogen evolution [120]. The cathodic performance of Ni_3_N-V_2_O_3_ was superior to only Ni_3_N or V_2_O_3_, and on par with Pt/C, as shown in Figure 26a. At the anode, Ni_3_N-V_2_O_3_ was similarly more active than Ni_3_N, and addition of 10 mM 5-HMF reduced overpotentials by about 0.14 V. Adding 5-HMF also caused the disappearance of oxidation peak of Ni^2+^ to Ni^3+^ before water splitting (Figure 26b). Experiments were performed using a two-electrode cell (both anode and cathode being Ni_3_N-V_2_O_3_) in 1 M KOH with 10 mM 5-HMF, at a fixed current of 10 mA/cm^2^ with corresponding overall potential of about 1.4 V. FDCA yield of 96.1% and selectivity of 98.7%, and hydrogen Faradaic efficiency of over 90% were reported, as shown in Figure 26c,d. 

Studies on evolution of hydrogen coupled with biomass electroreforming are summarized in Table 3 with the key technical information.

## 5. Conclusions and Outlook

The electroreforming of biomass compounds represents a promising green and sustainable route for synthesizing value-added chemicals with minimum damage to the environment. Compared to thermochemical routes, the operating conditions of electroreforming route are usually milder, and product controllability is better through tuning electrolytic cell parameters such as pH and potential. When compared to the biochemical routes, electroreforming processes can be conducted in more compact devices and in much shorter durations. For electroreforming of cellulose derivatives (glucose, 5-HMF, levulinic acid), exciting progress has been made by using various metal electrocatalysts to improve products and yields. In particular, recent studies have demonstrated the usage of non-noble metals or bimetallic alloys as electrocatalysts for both oxidation and hydrogenation processes. These developments shift reliance away from expensive noble metals, potentially increasing the economic viability of electroreforming techniques. Studies of flow reactor cells [54,67,98,108], showcased the possible continual production they provide, and therefore their industrial scalability. Notably, a great advantage of the electrochemical route is the cogeneration of green hydrogen, which plays an indispensable role in decarbonization. From an energy saving prospective, both holes and electrons from electricity are utilized in such hybrid electrolysis, coupling biomass electrooxidation and water reduction, leading to valuable cathodic and anodic products. Despite these promising advantages of biomass electroreforming over state-of-the-art biomass valorization, there are challenges to tackle before large-scale implementation.

Investigations into direct electroreforming of cellulose remains under-represented mainly due to the large polymer that could not be readily hydrolyzed in electrolyte. In order to make every stage in electroreforming pipeline green and sustainable, glucose and the other derivatives should be obtained from cellulose, so as not to compete with edible plant sources. At the time of writing, several studies have been published to uncover electrooxidation and depolymerization mechanisms of cellulose. A few studies have analyzed useful products from cellulose electrolysis [36]. Thus, more investigations into energy-efficient and cost-effective pre-treatment methods are needed for converting raw biomass polymers to smaller molecules that can be readily reformed by electrochemical process. To this end, the rational combination of mechanochemical and biological processes could potentially hold great promise.

High-value products would offset the overall production cost, increasing the economic viability of the electroreforming route. Raw biomass consists of large polymers and selectively converting them to high-value products is challenging. Ideally, one would like to get all possible reaction pathways and products mapped out, and then study how to control the selectivity. Therefore, in situ/operando measurements such as FTIR and SERS-based optical methods are promising approaches to shed light on the detailed reaction mechanisms. Moreover, complementary theoretical models to predict the thermodynamics and kinetics of the reaction are critical for a complete understanding of the reactions involved in electroreforming. Nevertheless, atomic modeling of large polymer necessitates superior computing facilities and is very costly. As studies performed by Roman et al. have shown, in situ methods such as spectroscopy can be combined with simulations for density functional theory calculations to provide enhanced understanding of reaction mechanisms [89]. A rational combination of in situ/operando characterization and theoretical modeling could lead to time- and energy-efficient investigation without compromising accuracy. 

Studies were also mostly focused on exploring the feasibility of different advanced catalysts and electrodes. It is noted that most reported catalysts show superior activity but inferior stability, which is very crucial for practical use. Many lessons can be learnt from the development of water electrolysis across the full pH range. Alkaline water electrolysis is by far the cheapest and most scalable technique for green hydrogen generation. Despite the recent drastic reduction in its cost, PEM water electrolysis still suffers from poor scalability mainly due to its Pt-group catalysts, particularly its anodic catalyst based on iridium and ruthenium. Similar challenge faces biomass electroreforming in acidic media. To this end, strategies for stabilizing non-precious catalyst and decreasing load of precious catalysts for PEM water electrolysis can be implemented for acidic biomass electroreforming. Nevertheless, biomass electroreforming in alkaline media is still relatively more cost-effective and scalable. Electroreforming of biomass represents a greener route of electrosynthesis of chemicals. Despite its advantage of better sustainability, it is challenging to control the reaction pathways. Advanced catalyst design, e.g., tandem catalysts, could enrich the toolbox of pathways for biomass electroreforming.

Lastly, in order to advance our collective understanding, forming a consistent benchmark on evaluating the efficacy of different designs will be critical. A standard protocol with critical parameters, such as potential, current density, and stability, for benchmarking is needed before one can compare the results across the literature. Such benchmarking will greatly facilitate the development of catalysts and electrodes. 

## Figures and Tables

**Table 1:** Studies investigating electroreforming of biomass at the anode.

Year	Electrocatalyst	Feedstock	Desired Products	Electrolyte	Electrolytic Tests and Conditions (Potentials Respect to RHE)	Yield/Output	Faradaic Efficiency	Reference
2010	polycrystalline Au	NaOH dissolved ball milled cellulose	Carboxyl groups	1.33 M NaOH	CV characterization (0.90 V to 1.40 V)	-	-	[34]
2015	Au nanoparticles/carbon aerogel	NaOH dissolved cellulose	Gluconate	0.125 M NaOH	10 mA/cm^2^ at 18 h	67.80%	-	[36]
2016	Au nanoparticles/carbon paper	NaOH dissolved cellulose	-	1.33 M NaOH	CV characterization (0.60 V to 1.40 V)	-	-	[41]
2011	Pb/PbO_2_	Cotton cellulose	Soluble sugars, 5-HMF	0.5 M H_2_SO_4_	30 mA/cm^2^ at 12 h	Soluble sugar: 2.5%, 5-HMF: 1.8%	-	[42]
2014	MnO_2_/Ti	Glucose	Glucaric and gluconic acids	0.07 M Na_2_SO_4_ (pH 7)	1.41 V at 19 min	99% (both glucaric and gluconic acids)	37.00%	[54]
2020	Polycrystalline Cu, Au, Pt	Glucose	Glucaric and gluconic acids	0.1 M NaOH	0.70 V at 65 h	selectivities up to 86.8% for gluconic acid, 13.5% for glucaric acid	-	[56]
2020	NiFeOx/NF, NiFeNx/NF, NiFe(OH)x/NF, NF, RuO_2_/NF, Pt-C/NF	Glucose	Glucaric and gluconic acids	1 M KOH	1.40 V at 18 h	11.6% (glucaric acid), 4.7% (gluconic acid)	87% (both)	[60]
2021	Pt_9_-Bi1/C, Pt/C	Glucose	Gluconic acid	0.1 M NaOH	0.30 V at 6 h	40% yield	~100.0%	[61]
2018	NiFe, NiAl, NiGa, NI(OH)2	5-hydroxymethylfurfural (5-HMF)	2,5-furandicarboxylic acid (FDCA)	1 M KOH	1.33 V at 10 h	98.00%	98.60%	[66]
2018	CoP, CoB, CoTe, Co_2_Si, CoAs on Ni foam	5-HMF	FDCA	1 M KOH	1.45 V at 70 min	94.00%	98.00%	[67]
2018	electrodeposited Cu/Cu foam	5-HMF	FDCA	0.1 M KOH	1.62 V at 8 h	96.40%	95.30%	[68]
2019	NiOOH, CoOOH, FeOOH/flouride doped tin oxide (FTO) glass	5-HMF	FDCA	0.1 M KOH	1.47 V at 4.7 h	96.00%	96.00%	[69]
2019	NiCo_2_O_4_, Co_3_O_4_/Ni foam	5-HMF	FDCA	1 M KOH	1.50 V at 53 min	90.40%	87.50%	[70]
2020	TpBpy-Ni@FTO	5-HMF	FDCA	0.1 M LiClO_4_	1.55 V at 40 min	58.00%	-	[71]
2020	CoO-CoSe_2_	5-HMF	FDCA	1 M KOH	1.43 V at 1 h	99.00%	97.90%	[72]
2021	WO3 on Ni foam	5-HMF	FDCA	1 M KOH	1.57 V at 351 min	81.50%	79.50%	[73]
2021	Ni3S2/NF, NiS, NiO, NiC, NF	5-HMF	FDCA	1 M KOH	1.50 V at 2 h	98.30%	93.50%	[74]
2018	MnOx/FTO glass	5-HMF	FDCA and maleic acid	0.05 H_2_SO_4_	1.60 V (duration not reported)	53.8% FDCA, 21.9% maleic acid	33.8% (FDCA)	[75]
2015	Pt, C, Cu, Fe, Ni, Pb	Levulinic acid	Valeric acid, g-valerolactone, 4-hydroxy-2-butanone and other hydrocarbons	0.5 M H_2_SO_4_ (C for g-valerolactone and Pb for valeric acid), 0.2 M NaOH (C for 4-hydroxy-2-butanone)	−1.80 V (g-valerolactone and valeric acid), 6.00 V (4-hydroxy-2-butanone), at 4 to 8 h	27.2% for g-valerolactone, ~56% for valeric acid, 6% selectivity for 4-hydroxy-2-butanone	60.0% for valeric acid, 18.0% for g-valerolactone, 5.0% for 4-hydroxy-2-butanone	[76]
2020	CuO	Glycerol	1,3-dihydroxyacetone	0.1 M Na_2_B_4_O_7_	3 mA/cm^2^ at 3 h	selectivity of 60.0%	-	[82]
2021	CoOx	Glycerol	1,3-dihydroxyacetone	0.1 M Na_2_B_4_O_7_	1.50 V at 3 h	selectivity of 60.0%	49.40%	[84]
2015	Bi-Pt/C, Sb-Pt/C	Sorbitol	Varied	0.5 M H_2_SO_4_	CV characterization (0 to 1.60 V)	-	-	[85]
2018	PbO_2_, MnO_2_, Pt	Furfural	Maleic acid	H_2_SO_4_ (pH of 1)	2.00 V (duration not reported)	65.10%	-	[88]
2020	Au/Carbon cloth	Furfural	Furoic acid	0.25 M HClO_4_	CV characterization (0 to 1.50 V)	-	-	[89]

**Table 2:** Studies investigating electroreforming of biomass at the cathode.

Year	Electrocatalyst	Feedstock	Products	Electrolyte	Electrolytic Tests and Conditions (Potentials Respect to RHE)	Yield	Faradaic Efficiency	Reference
2014	MnO_2_/graphite/PTFE	short chain cellulose oligosaccharides	Glucose	0.1 M Na_2_SO_4_ (pH of 3)	−0.58 V at 8 h	72.40%	-	[90]
2013	Cu, Ni, Pt, Pb, C, Al	5-HMF	2,5-dimethylfuran (DMF)	0.5 M H_2_SO_4_	10 mA/cm^2^, 2 to 4 h	selectivity of 35.6%	up to 67%	[87]
2013	Fe, Ni, Ag, Cd, In, Au, Sn, Sb, Co, Bi, Pd, Pb	5-HMF	1,5-dihydroxymethylfuran (DHMF)	0.1 M Na_2_SO_4_ (pH of 7)	CV characterization for different electrodes	-	-	[91]
2015	Pd, Pt, Al, Zn, In, Sb, Co, Ag, Cd, Bi	5-HMF	DHMF	0.5 M H_2_SO_4_	CV characterization for different electrodes	-	-	[92]
2019	Ag/C	5-HMF	2,5-bis(hydroxymethylfuran) (BHMF)	0.5 M borate buffer (pH of 9)	−0.46 at 30 min, paired with TEMPO mediated 5-HMF oxidation	15.9%	89.3% (cathode)	[96]
2019	CuNi, Cu, Ni	5-HMF	DMF	0.2 M Sulfate buffer (pH of 2)	−0.48 V (duration not reported)	34.00%	84.60%	[97]
2021	Oxide Derived (OD) Ag	5-HMF	BHMF	0.5 M borate buffer (pH of 9)	−0.51 V at 3 h, paired with TEMPO 5-HMF	28.60%	~80% (cathode)	[98]
2012	Pb	Levulinic acid	Valeric acid (ECH), octane (Kolbe)	0.5 M H_2_SO_4_ (ECH), water or methanol at pH of 5.5 (Kolbe)	−1.41 V at 4 h (ECH), 3.895 V (duration unspecified) (Kolbe)	Valeric acid selectivity 97.2%, octane selectivity 51.6%	27% (ECH), 66.5% (Kolbe)	[77]
2013	Cu, Pb	Levulinic acid	Valeric acid, g-valerolactone	0.5 M H_2_SO_4_ (pH of 0), K_2_HPO_4_ buffer (pH of 7.5)	−1.10 V to −1.50 V, 4 h	Valeric acid maximum yield ~12%, g-valerolactone max yield ~1%	-	[104]
2015	C, Cu, Ni, Pb, Fe	Levulinic acid	g-valerolactone	Varied (acidic, neutral, alkaline)	−1.80 V, 4 to 8 h	valeric acid 56.0% (Pb acidic), g-valerolactone 28.0% (Fe alkaline)	~20%	[76]
2020	Pb, Pt, Zn, Cu, Co, Ti	Levulinic acid	Valeric acid	0.5 M H_2_SO_4_	−1.40 V at 4 h	43.20%	94.00%	[107]
2021	Pt	Levulinic acid	1,2-octanedione	0.1 M KOH	-	75.00%	67.00%	[108]

**Table 3:** Studies investigating evolution of hydrogen coupled with biomass electroreforming.

Year	Electrocatalyst	Feedstock	Desired Product(s)	Electrolyte (Catholyte)	Electrolytic Conditions	Coupled Product Yield (If Any)	OER Potential	Reactant Oxidation Potential	Reference
2017	Fe_2_P/Stainless steel mesh	0.5 M glucose	Hydrogen	10 M KOH	CV characterization	-	1.52 V for 10 mA/cm^2^	1.22 V for 10 mA/cm^2^	[109]
2019	Pd-Au/C	0.1 M glucose	Hydrogen, gluconic acid	0.1 M NaOH	CV characterization, applied 0.40 V at 6 h	Gluconate yield 58.3%	-	0.2 V for 1 mA/cm^2^, 0.5 V for max 4 mA/cm^2^	[110]
2020	Co, Ni, Co-Ni/Carbon cloth	0.1 M glucose	Hydrogen	1 M KOH	CV characterization	-	1.39 V for 10 mA/cm^2^	1.096 V for 10 mA/cm^2^	[112]
2020	Co_0.5_ Ni_0.5_(OH)_2_ Nanosheet	0.1 M glucose	Hydrogen	1 M KOH	CV characterization	-	1.47 V for 10 mA/cm^2^	1.22 V for 10 mA/cm^2^	[113]
2020	Ni-MoS_2_/carbon paper	0.3 M glucose	Hydrogen	1 M KOH	CV characterization	-	1.64 V for 10 mA/cm^2^	1.46 V for 10 mA/cm^2^	[114]
2020	Fe_0.1_-CoSe_2_/Carbon cloth	0.5 M glucose	Hydrogen, gluconic acid	0.5 M H_2_SO_4_	CV characterization	-	1.34 V for 10 mA/cm^2^	0.72 V for 10 mA/cm^2^	[115]
2020	glucose derived carbon/glassy carbon	sacrificial carbon at cathode	Hydrogen	0.1 M KOH	CV characterization	-	OER onset 1.52 V	Carbon oxidation onset 1.02 V	[116]
2021	Mo-Ni0.85Se/NF	10 mM 5-HMF	Hydrogen, FDCA	1 M KOH	1.40 V at 2 h	FDCA yield 95.0%, FE 95.0%	Overall 1.68 V for 50 mA/cm^2^	Overall 1.5 V for 50 mA/cm^2^	[117]
2016	Co-P/Cu foam	50 mM 5-HMF	Hydrogen, FDCA	1 M KOH	1.42 V at 6 h	FDCA yield 90.0%	Anodic 1.53 V, overall 1.59 V for 20 mA/cm^2^	anodic 1.38 V, overall 1.44 V for 20 mA/cm^2^	[118]
2021	Ni_3_N-V_2_O_3_	10 mM 5-HMF	Hydrogen, FDCA	1 M KOH	10 mA/cm^2^ at 112 min	FDCA yield 96.1%	-	1.4 V for 10 mA/cm^2^	[120]
